# An Egg White-Derived Peptide Enhances Systemic Insulin Sensitivity and Modulates Markers of Non-Alcoholic Fatty Liver Disease in Obese, Insulin Resistant Mice

**DOI:** 10.3390/metabo13020174

**Published:** 2023-01-24

**Authors:** Stepheny C. de Campos Zani, Ren Wang, Hellen Veida-Silva, Robin D. Clugston, Jessica T. Y. Yue, Marcelo A. Mori, Jianping Wu, Catherine B. Chan

**Affiliations:** 1 Department of Physiology, University of Alberta, Edmonton, AB T6G 2H7, Canada; 2 Alberta Diabetes Institute, University of Alberta, Edmonton, AB T6G 2E1, Canada; 3 Department of Agricultural Food & Nutritional Science, University of Alberta, Edmonton, AB T6G 1C9, Canada; 4 Molecular and Cell Biology of Lipids Group, University of Alberta, Edmonton, AB T6G 2H7, Canada; 5 Department of Biochemistry and Tissue biology, University of Campinas, Campinas P.O. Box 6109, Brazil

**Keywords:** bioactive peptides, egg, metabolic syndrome, non-alcoholic fatty liver disease, type 2 diabetes

## Abstract

Non-alcoholic fatty liver disease (NAFLD), the hepatic manifestation of the metabolic syndrome, is a global health problem. Currently, no pharmacological treatment is approved for NAFLD. Natural health products, including bioactive peptides, are potential candidates to aid in the management of metabolic syndrome-related conditions, including insulin resistance and obesity. In this study, we hypothesized that an egg-white-derived bioactive peptide QAMPFRVTEQE (Peptide 2) would improve systemic and local white adipose tissue insulin sensitivity, thereby preventing high-fat diet-induced exacerbation of pathological features associated with NAFLD, such as lipid droplet size and number, inflammation, and hepatocyte hypertrophy in high-fat diet-fed mice. Similar to rosiglitazone, Peptide 2 supplementation improved systemic insulin resistance during the hyperinsulinemic-euglycemic clamp and enhanced insulin signalling in white adipose tissue, modulating ex vivo lipolysis. In the liver, compared with high-fat diet fed animals, Peptide 2 supplemented animals presented decreased hepatic cholesterol accumulation (*p* < 0.05) and area of individual hepatic lipid droplet by around 50% (*p* = 0.09) and reduced hepatic inflammatory infiltration (*p* < 0.05) whereas rosiglitazone exacerbated steatosis. In conclusion, Peptide 2 supplementation improved insulin sensitivity and decreased hepatic steatosis, unlike the insulin-sensitizing drug rosiglitazone.

## 1. Introduction

Metabolic syndrome pathophysiology exemplifies a clear crosstalk between major metabolic organs, including the liver and white adipose tissue (WAT). Non-alcoholic fatty liver disease (NAFLD) affects 25% of the global population and is strongly associated with obesity, type 2 diabetes (T2D)/insulin resistance (IR), and dyslipidemia. All of these conditions are a public health concern and beget socioeconomic problems [1]. Hepatic steatosis in NAFLD results from an imbalance between substrate availability (fatty acids and carbohydrates) and the hepatic capacity to dispose of fats properly. In humans, the two main sources of non-esterified fatty acids (NEFA) in the liver are WAT lipolysis and de novo lipogenesis (DNL) [2]. DNL produces fatty acids from non-lipid precursors such as glucose or fructose and is increased in IR states [3], and plays an important role in NAFLD [4]. NAFLD may progress to non-alcoholic steatohepatitis (NASH) in which inflammation, fibrosis and cellular damage are present, then to cirrhosis and further to hepatic cancer, increasing the need for liver transplantation [2] and seriously impacting quality of life.

Lifestyle interventions (diet and physical activity) improve NAFLD, but currently no pharmacological treatment is approved for NAFLD. Several drugs, including thiazolidinediones (peroxisome proliferator-activated receptor gamma (PPARγ) agonists that are insulin sensitizers) are being investigated as therapies to reduce hepatic steatosis, inflammation, and fibrosis [5,6]. However, findings are controversial [7,8]. Natural health products and functional foods include potential candidates to aid in the management of metabolic conditions. Food-derived bioactive peptides have effects beyond their nutritive value and can modulate physiological processes promoting health benefits [9]. There are many food sources of bioactive peptides, including the egg, a universally available and consumed source of protein.

Previously, our group showed that an egg white hydrolysate (EWH) alleviates hypertension [10] and IR in rat models [11], and mimics insulin action in preadipocytes [12]. In addition, IRW, a specific peptide found in the EWH improves hypertension [13] and IR in rodents [14]. Another peptide identified in the EWH is QAMPFRVTEQE (aka Peptide 2), which mimics insulin actions to enhance PPARγ protein abundance and other markers of adipogenesis in preadipocyte cell culture [15] but its in vivo efficacy is not established. Considering the need of new therapies for NAFLD and the crosstalk between insulin signaling, WAT and the liver, we aimed to identify specific effects of Peptide 2 diet supplementation on manifestations of the metabolic syndrome including systemic IR, WAT response to insulin and NAFLD markers, compared with the thiazolidinedione rosiglitazone. We hypothesized that Peptide 2 supplementation improves systemic and local insulin sensitivity, which in turn alleviates pathological cellular features associated with NAFLD, therefore modulating both glucose and lipid metabolism.

## 2. Materials and Methods

### 2.1. Animals and Diet

Protocol 1: This protocol and some results of a previous intervention trial were previously published by our group [11]. Briefly, male Sprague Dawley (SD) rats (n = 48) were fed with high fat diet (HFD) for 6 wks. Then, half of the animals received HFD+4% EWH with the remainder serving as HFD controls for another 6 wks. At the end of week 12, half of the animals received an intraperitoneal injection of insulin (2 IU/kg of body weight (BW)) to stimulate insulin signaling prior to euthanization using CO_2_. Diet composition was published elsewhere [11] and was matched for macronutrient and energy content. Herein, we report lipolysis pathway data from WAT tissues; a full description of the rat phenotype after EWH treatment is published elsewhere [11].

Protocol 2: Male C57BL/6 mice (5 wks old) purchased from Charles River Canada were housed 4/cage with ad libitum access to food and water, exposed to 12:12 h light:dark in a humidity- and temperature-controlled environment (60% humidity, 23 °C). Mice received a low fat diet (LFD, 10% kcal fat) or a high fat diet (HFD, 45% kcal fat) for 6 wks. After that, the HFD animals were divided into 3 groups: HFD only, HFD + Peptide 2 (PEP2) and HFD + rosiglitazone (ROSI) and continued receiving their respective diets for another 8 wks. LFD animals continued receiving LFD for another 8 wks. After a total of 14 wks, mice either received an intraperitoneal injection of insulin (1.5 IU/kg BW) prior to euthanasia or were directly euthanized using CO_2_, while some mice underwent hyperinsulinemic-euglycemic clamp prior to euthanasia by ketamine. Diet composition is shown in Table S1. Peptide 2 was administered at 45 mg/Kg BW/day daily mixed in the diet. The characteristics of Peptide 2 are reported in Table 1 [15]; it was synthesized by Genscript (Piscataway, NJ, USA) with 97.9% compound purity and no terminus modifications. Peptide 2 is soluble in water, dimethyl sulfoxide and phosphate buffered saline at a concentration ≤10 mg/mL. High performance liquid chromatography chromatogram and the mass spectra of the peptide provided by Genscript are shown in Figure S1. Rosiglitazone (Sigma-Aldrich, ST. Louis, MA, USA) was administered at 2.5 μmol/kg BW/day in the drinking water.

### 2.2. Body Weight, Body Composition and Sample Collection

Mice were weighed weekly. Body composition was measured at week 14 in fasted conditions using an ECHO magnetic resonance imaging (ECHO MRI) as per manufacturer’s instructions. Blood was collected by cardiac puncture into EDTA tubes and plasma was stored at −80 °C until further analysis. Liver and WAT (retroperitoneal (rWAT), epidydimal (eWAT) and inguinal (iWAT)) were collected, weighed and snap frozen in liquid nitrogen. A sample of each tissue was fixed in formalin, dehydrated and preserved in paraffin blocks for histological analysis.

### 2.3. Adipose Tissue Organ Culture

During tissue collection, a piece of approx. 100 mg each and of each fat pad (iWAT, eWAT and rWAT) were collected and washed with cold phosphate-buffered saline + 1% penicillin/streptomycin (Gibco, Waltham, MA, USA) and kept in M199 media (Sigma-Aldrich, St. Louis, MA, USA) supplemented with 50 µU insulin (Sigma-Aldrich, St. Louis, MA, USA), 2.5 nM dexamethasone (Sigma-Aldrich, St. Louis, MA, USA) and 1% penicillin/streptomycin in a cell incubator at 37 °C for 24 h. After that, the media was replaced with fresh M199 supplemented only with 2.5% fatty acid-free bovine serum albumin (MP Biomedicals, St Ana, CA, USA). Each piece received one of the following treatments: sterile H_2_O or norepinephrine (1 uM, Sigma-Aldrich, St. Louis, MA, USA) or norepinephrine (1 uM) + insulin (1 IU/mL) for 2 h. An aliquot of the media was collected after 2 h and kept at −80 °C for future glycerol analysis.

### 2.4. Preadipocyte Cell Culture

Preadipocytes derived from mouse inguinal WAT (9 W) and from brown adipose tissue (9 B) were cultured and differentiated as previously described [16]. Briefly, cells were cultured in Dulbecco’s modified Eagle’s medium (DMEM) containing 10% of fetal bovine serum and 1% penicillin/streptomycin until confluence was reached. After that, cells were differentiated in DMEM containing 20 nM insulin, 1 nM triiodothyronine, 0.5 mM isobutyl methylxanthine, 1 μM dexamethasone, 0.125 mM indomethacin, and 2.8 μM of rosiglitazone. Because we wanted to compared the effect of Peptide 2 with rosiglitazone during preadipocyte differentiation, we modified the above differentiation cocktail as follows: the control (C) received the cocktail described above, the C+PEP2 was treated with the above cocktail supplemented with 100 μM of Peptide 2, the negative control (Rosi neg) received the above cocktail without rosiglitazone and the Rosi neg+PEP2 received the above cocktail without rosiglitazone but supplemented with Peptide 2 (100 μM).

### 2.5. Hyperinsulinemic-Euglycemic Clamp

The euglycemic clamp was performed as previously described [17,18] with the following modifications: briefly, mice were anaesthetized using ketamine (90 mg/kg BW) and xylazine (10 mg/kg BW) and underwent aseptic right jugular vein catheterization for intravenous infusions. Post-surgical body weight and food intake were monitored daily. After 3–4 days (to re-establish a minimum of 90% of pre-surgical BW), the mice were fasted for 5 h and underwent a hyperinsulinemic-euglycemic clamp, in which a primed, continuous infusion of tritiated glucose (1 μCi bolus + 0.1 μCi infusion; Perkin Elmer, Waltham, MA, USA) was maintained for the duration of the experiment to assess glucose kinetics. After a basal period, the hyperinsulinemic-euglycemic clamp was initiated with a primed, continuous infusion of insulin (3.0 mU/kg/min) for 120 min, and plasma glucose levels were maintained at a similar euglycemic level to the basal period using a variable infusion of 10% glucose solution. Plasma samples were obtained every 10 min for the measurement of glucose concentration (Analox Glucose Analyzer, Huntington beach, CA, USA) and [3-3H]-glucose specific activity. At conclusion of the clamp period, mice were euthanized using an infusion of 0.02 mL ketamine via the jugular vein, followed by decapitation.

### 2.6. Oral Glucose Tolerance Test (OGTT)

OGTT were performed at week 13. Briefly, after overnight fasting a bolus of glucose (1 g/kg BW) was orally gavaged to mice and blood glucose was measured after 0, 15, 30, 60, 90, and 120 min from the tail vein using a glucometer (Contour^®^ Next, Mississauga, ON, CA). OGTT were performed at week 14. After 4 hr fasting, mice received an intraperitoneal injection with insulin (0.75 U/Kg BW) and glucose was measured after 0, 15, 30, 60, 90, and 120 min as cited above.

### 2.7. Liver Triglyceride and Cholesterol Content

Liver triglyceride (TG) and cholesterol were extracted using approximately 100 mg of tissue and as previously described [19]. Briefly, tissue was homogenized in 1 mL of NaCl solution. A total of 500 uL of the extract was mixed with 2 mL of Folch solution (chloroform:methanol (2:1)), centrifuged at 3000× *g* rpm for 10 min and the lower phase collected. Samples were dried under nitrogen and resuspended with 1 mL of 2% TritonX-100 solution in chloroform and dried under nitrogen. The dried sample was then resuspended in ddH_2_O and kept at −20 °C until further use. Triglyceride and cholesterol content was measured using a commercial kit (Infinity^TM^, Thermo Scientific, Waltham, MA, USA).

### 2.8. PPARγ DNA Binding Activity

Nuclear protein extraction was performed using a commercial kit (Active Motif Inc., Carlsbad, CA, USA) following the manufacturer’s instructions for frozen tissue and using 100 mg of tissue for each extraction. PPAR-γ DNA binding activity was assessed by a TransAM^TM^ PPAR-γ kit (Active Motif Inc., Carlsbad, CA, USA) using 10 μg/10μL of nuclear protein extract following the manufacturer’s instructions.

### 2.9. Plasma Biochemical Analysis

All biochemical parameters were assessed after overnight fasting (14–16 h) and using the following commercial kits or reagents as per manufacturer’s instructions: mouse insulin ELISA (ALPCO, Salem, NH, USA); NEFA and liver L-type triglyceride M colorimetric assay (Wako Pure Chemical Industries Ltd., Richmond, VA, USA); adiponectin and resistin (Mesoscale Discovery); non-esterified fatty acids using glycerol free reagent as standard (Sigma-Aldrich, St. Louis, MA, USA); alanine transaminase (ALT) (Abcam, Cambridge, UK). Homeostatic model assessment of insulin resistance (HOMA-IR) was calculated using the formula: [fasting glucose (mmol/L)] × [fasting insulin (μU/L)]/22.5].

### 2.10. Protein Extraction and Western Blot

All reagents were purchased from Sigma-Aldrich (Sigma-Aldrich, St. Louis, MA, USA) unless otherwise specified. Liver tissue was homogenized using a tissue homogenizer in RIPA buffer (50 mM Tris HCL pH:8.0, 150 mM NaCl, 0.1% Triton X-100, 0.5% sodium deoxycholate, 0.1% SDS) supplemented with 2 µg/mL aprotinin (Calbiochem), 5 mM sodium fluoride, 5 mM sodium orthovanadate, and protease inhibitor cocktail (FastPrep^®^-24, MP Biomedicals). Adipose tissue protein was extracted using an extraction kit (AT-022, Invent biotechnologies, Plymouth, MN, USA). Lysates were stored at −80 °C for future analysis. Protein extracts were separated by SDS-PAGE 12% polyacrylamide gels as previously reported [11] and probed for p-AKT (Cell Signaling Technology (CS-4060S), AKT (CS-9272), PPARγ (Santa Cruz Biotechnology-7196), AT2R (abcam92445), p-HSL (CS-41265), HSL (abcam45422), p-PKA (CS-5661S or PKA (CS-58425) overnight before incubation with fluorescent secondary antibodies (Li-cor Biosciences) for 1 h at RT. Images were analyzed using Image Studio Lite software (Li-cor Biosciences, Lincoln, NE, USA). All the phosphorylated proteins bands were normalized to their corresponding total protein. Total proteins were normalized to β-actin (Sigma-Aldrich A5441).

### 2.11. Histology

Paraffin blocks of liver or WAT were cut into 5 μm sections and affixed to glass slides. Hematoxylin and eosin (H&E) staining was performed as previously reported [11]. Fibrosis was assessed using Masson’s trichrome staining kit (Sigma-Aldrich, St. Louis, MA, USA). Adipocyte size: 10 random photomicroscopic images of each slide (1 slide per animal) were captured using the microscope 20× objective lens and Axion Vision 4.8 software. ImageJ software “freehand selections” tool was used to measure adipocyte area (mm^2^) of 300 cells or 10 images per sample, whichever was reached first. Liver morphological characterization: Random photomicroscopic images (20×, Axio Vision 4.8 software, n = 3 per mouse) were taken and a researcher blinded to group assignment used ImageJ software “freehand selections” tool to quantify lipid droplet (LD) area, cell number and inflammatory foci (a cluster with >5 immune cells). Each image was divided into four equal areas and the top left quadrant (standard area: 88,884.66 μm^2^) was analyzed as a representation of the total image. In terms of LD size, there is not a defined numerical threshold for small or large LD categories. However, based on the literature, hepatic lipid accumulation was divided into three categories, macrovesicular with one large LD displacing the nucleus to the side, macrovesicular with one single small LD not displacing the nucleus and true microvesicular steatosis where several small LD occupy a hepatocyte, giving it a foamy appearance [20,21,22].

### 2.12. Quantitative PCR (qPCR)

Primer sequences are provided in Table S2. Liver RNA was extracted using the QIAGEN RNeasy min plus kit following the manufacturer’s instructions and as previously described [23] with the following modifications: frozen tissue (50–100 mg) was lysed and homogenized using 1 mL of TRIzol. After 5 min at RT, 0.2 mL of chloroform per mL of TRIzol was added. Samples were shaken vigorously and incubated at RT for 3 min, followed by centrifugation at 12,000× *g* for 10 min at 2–8 °C. The supernatant was collected, and the manufacturer’s instructions were followed for the remaining steps until RNA was obtained. RNA concentration and purity were measured using a Nanodrop (Thermo Fisher, Waltham, MA, USA) and cDNA synthesis was performed using the high-capacity cDNA RT kit (Applied Biosystems, Waltham, MA, USA) using 2 μg RNA per reaction in a ProFlex PCR system thermo cycler (Applied Biosystems, Waltham, MA, USA). qPCR was performed using PerfeCTa SYBR Green SuperMix ROX (Quantabio, Beverly, MA, USA) in a QuantStudie3 machine (Applied Biosystems, Waltham, MA, USA) using beta actin as the reference gene.

### 2.13. Statistical Analysis

All data presented are expressed as means ± SEM of ‘n’ mice as indicated in each figure description. Statistical analysis was performed using GraphPad Prism software 7.0 (GraphPad Software Inc., San Diego, CA, USA). Data were checked for normal distribution by the Shapiro–Wilk test and any identified outliers were removed. T-test was used to compare LFD to HFD (to establish IR-related differences), while one-way ANOVA was used to compare HFD groups (i.e., HFD, PEP2 and ROSI) to identify treatment effects. Two-way ANOVA was used to compare insulin regulation of AKT, PKA and HSL. Bonferroni’s or Dunn’s post-hoc tests were performed to assess differences between groups when a significant main effect was observed. A *p*-value ≤ 0.05 was considered statistically significant.

## 3. Results

### 3.1. EHW Effects on WAT Lipolytic Pathway from Obese, Insulin Resistant Rats

**Figure 1:**
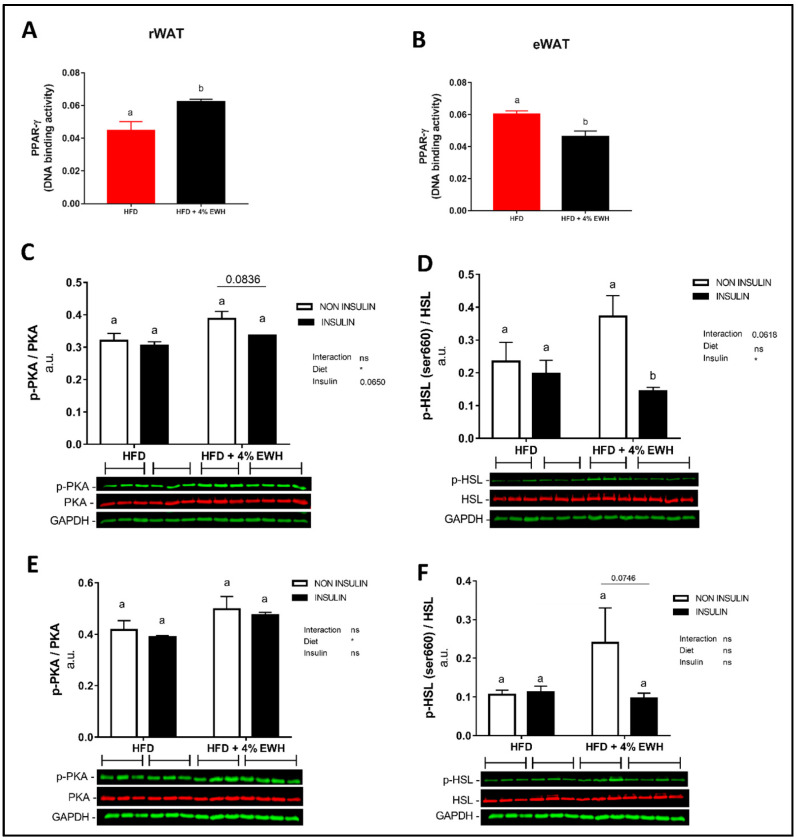
EWH effects in WAT of Sprague Dawley rats fed EWH for 6 weeks (Protocol 1). PPARγ DNA binding activity in rWAT (**A**) and eWAT (**B**). Data expressed as mean ± SEM and analyzed by two-tailed t-test (n = 6–7). PKA, p-PKA, HSL and p-HSL protein abundance in rWAT (**C**,**D**) and eWAT (**E**,**F**). Data expressed as mean ± SEM and analyzed by two-way ANOVA (n = 3–4). Bars with different letters indicate *p* < 0.05. EWH, egg white hydrolysate; PKA, protein kinase A; PPARγ, Peroxisome proliferator-activated receptor gamma; HSL, hormone sensitive lipase; WAT, white adipose tissue; rWAT, retroperitoneal WAT; eWAT, epididymal WAT.

Previously we showed that 4% EWH improved glucose tolerance and insulin sensitivity, reduced adipocyte size and enhanced PPARγ abundance in WAT in rats [11]; thus, its effects on lipolytic enzymes in WAT was investigated here. PPARγ DNA binding activity was reduced by 4% EWH in eWAT (*p* < 0.01), but significantly increased in rWAT (*p* < 0.01) (Figure 1A,B). The investigation of key enzymes involved in lipolysis by two-way ANOVA showed a significant overall diet effect (*p* < 0.05) in rWAT p-PKA/PKA ratio and an overall insulin effect (*p* < 0.05) in p-HSL/HSL. The post-hoc analysis revealed that 4% EHW treated animals had reduced p-HSL in rWAT after intraperitoneal injection of insulin (*p* < 0.05) despite no change in phosphorylation on PKA (Figure 1C,D). No changes in total PKA or HSL protein abundance were seen in rWAT (Figure S2). In eWAT, two-way ANOVA revealed a significant overall diet effect (*p* < 0.05) on p-PKA/PKA, while no overall effect was seen in p-HSL/HSL. No changes in p-HSL or p-PKA (Figure 1E,F) or total HSL (Figure S2) were seen. However, total PKA abundance was reduced by 4% EWH treatment (Figure S2). Plasma and WAT adiponectin and resistin concentrations were not different between groups (Table 2).

Based on these indications that 4% EWH had the potential to improve insulin-mediated suppression of lipolysis and activate PPARγ, a trial of 4 EWH-derived, purified peptides (Peptides 1–4) that elicited increased PPARγ in vitro [15] was initiated. From preliminary data (n = 12 mice/group), insulin tolerance was improved by Peptide 2 (*p* < 0.05) together with lower iWAT and rWAT weights compared to HFD (Figure S3), whereas Peptides 1, 3 and 4 did not affect any WAT depot weight. Notably, Peptide 2 had the lowest and ROSI the highest liver weight. Therefore, additional experiments were performed, focusing on the effects of Peptide 2 on the IR phenotype and iWAT/rWAT lipid metabolism.

### 3.2. Peptide 2 and Rosiglitazone Effects in HFD Induced Obese and Insulin Resistant Mice

#### 3.2.1. Food Intake, Body Composition and Tissue Weight

Food and water intake were not different between any of the groups compared to HFD animals (Figure S4A–D). As expected, after 6 wks of HFD feeding, the HFD group presented higher BW than LFD animals (Table 3) and were glucose intolerant (Figure S5). At the end of the trial, HFD group maintained higher BW and BW gain than LFD. Initial BW was not different between all the HFD groups; however, the ROSI group had reduced rate of BW gain than HFD group after 3 weeks of treatment leading to a reduced final BW. Peptide 2 supplementation did not influence final BW or BW gain in comparison to HFD. Body composition analysis revealed that only the LFD animals had a lower fat mass % and higher lean mass % than HFD (Table 3).

Compared to HFD, LFD animals had decreased mass of all three fat pads. ROSI group presented lower rWAT mass compared to HFD, while PEP2 animals had an intermediate rWAT mass, between ROSI and HFD groups. eWAT and iWAT did not change between all the HFD groups. Liver weight of LFD and HFD groups was not different. ROSI animals had a heavier liver than all the other HFD groups (Table 3).

#### 3.2.2. Plasma Biochemical Parameters

Fasted LFD animals had lower blood glucose concentration than HFD, while no statistical difference was seen between the HFD groups. No statistical difference was seen in fasting plasma insulin concentration between any of the groups, despite a considerable reduction in LFD (*p* < 0.1) and ROSI groups compared to HFD. This was accompanied by a lower HOMA-IR in LFD animals compared to HFD but no differences between HFD groups. No changes were seen regarding plasma lipid profile (NEFA and TG) between any of the groups (Table 3).

#### 3.2.3. Glucose Homeostasis and Systemic Insulin Sensitivity

**Figure 2:**
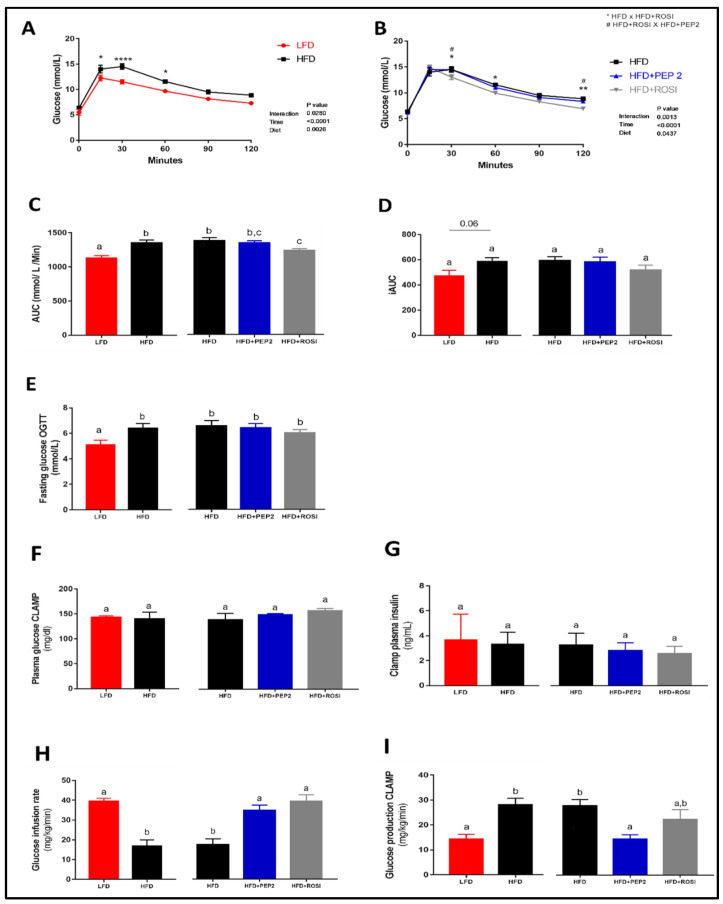
Glucose tolerance test and hyperinsulinemic-euglycemic clamp in mice treated with Peptide 2 (Protocol 2). (**A**,**B**) Glucose tolerance test (OGTT), (**C**) OGTT area under the curve (AUC), (**D**) OGTT incremental AUC, (**E**) overnight fasting glucose on OGTT day (n = 11–12). Data expressed as mean ± SEM and analyzed by two-tailed t-test (LFD vs. HFD) and by one-way ANOVA or Kruskal-Wallis (HFD groups) or two-way ANOVA. In the hyperinsulinemic-euglycemic clamp: (**F**) plasma glucose concentration; (**G**) plasma insulin; (**H**) glucose infusion rate and (**I**) glucose production. Data expressed as mean ± SEM of n = 4–7 and analyzed by two-tailed t-test (LFD vs. HFD) or by one-way ANOVA (HFD groups). Bars with different letters indicate *p* < 0.05. * and # indicate *p* < 0.05; ** indicates *p* < 0.01; **** indicates *p* < 0.0001.

In vivo tests confirmed that at week 14 HFD animals were glucose intolerant compared to LFD animals (Figure 2A,C), and that the ROSI group had improved glucose tolerance compared to HFD group (Figure 2B,C, right). Two-way ANOVA analysis show a significant treatment × time interaction (*p* < 0.0001) and a significant effect of diet (*p* = 0.0437) among the HFD groups. However, despite changes in the OGTT curve and AUC, the incremental AUC was not different between groups (Figure 2D). This is likely because fasting glucose concentration in the OGTT was significantly lower in the LFD group compared to HFD (Figure 2E, left). During the hyperinsulinemic-euglycemic clamp LFD, ROSI and PEP2 groups had improved insulin sensitivity compared to HFD (Figure 2F–I and Figure S6). Plasma glucose during the hyperinsulinemic-euglycemic clamp was not different among the groups (Figure 2F). Similarly, there was no difference in plasma insulin during the clamp procedure (Figure 2G), both validating the clamp technique. IR of the HFD group was indicated by the lower glucose infusion rate (GIR) (Figure 2H, left) and higher glucose production during the clamp than LFD (Figure 2I, left). Moreover, GIR was higher in PEP2 and ROSI compared to HFD (Figure 2H, right). In addition, glucose production was reduced in the PEP2 group compared to HFD, while ROSI showed an intermediate effect (Figure 2I, right). When comparing basal vs. clamp glucose production within group, glucose production was only suppressed in the PEP2 group, with a similar pattern in the LFD group (Figure S6A). Insulin-stimulated glucose disposal was higher in the LFD and ROSI groups compared to HFD and although it was also numerically higher in the PEP2 group, no significance was seen (Figure S6B). Improvement in insulin sensitivity after PEP2 and ROSI treatment was confirmed during the insulin tolerance test (Figure S6C–I).

#### 3.2.4. WAT Regulation by Insulin: Lipolysis and AKT

**Figure 3:**
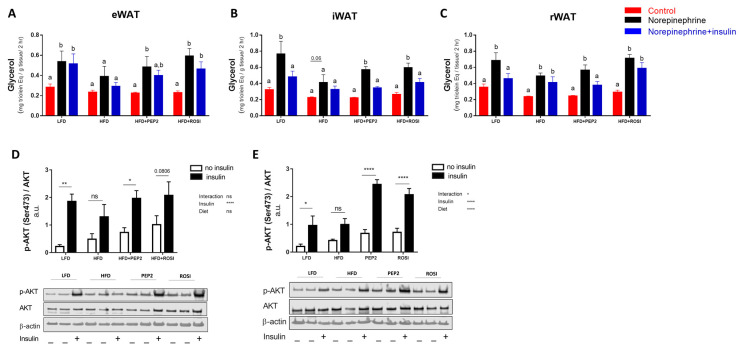
White adipose tissue (WAT) ex vivo lipolysis and protein kinase B (AKT) activation in tissues harvested from mice treated with Peptide 2 (Protocol 2). Lipolysis ex-vivo in (**A**) eWAT (n = 5–6), (**B**) iWAT (n = 5–6) and (**C**) rWAT (n = 4–6). (**D**) rWAT p-AKT/AKT (n = 6) and (**E**) iWAT p-AKT/AKT (n = 6). Data expressed as mean ± SEM and analyzed by two-way ANOVA (**D**,**E**). Bars with different letters and * indicate *p* < 0.05; ** *p* < 0.01 and **** *p* < 0.0001. HFD, high fat diet; rWAT, retroperitoneal WAT; eWAT, epididymal WAT, iWAT inguinal WAT; LFD, low fat diet; ROSI, rosiglitazone.

Tissue collected from fasted HFD animals had impaired norepinephrine-stimulated lipolysis ex vivo in eWAT and iWAT (Figure 3A,B), while in rWAT lipolysis stimulation occurred in all the groups, despite HFD having a lower magnitude of stimulation (Figure 3C). Two-way ANOVA diet overall effect was significant in rWAT and iWAT (*p* < 0.05), but not in eWAT. The overall stimulatory effect was significant in all three fat pads (*p* < 0.05). Interestingly, none of the groups showed suppression of lipolysis by insulin in the eWAT (Figure 3A), but the lack of suppression of lipolysis by insulin in HFD was rescued by Peptide 2 in both iWAT (Figure 3B) and rWAT (Figure 3C). ROSI normalized lipolysis suppression in iWAT (Figure 3B) but not in rWAT (Figure 3C). Because only rWAT and iWAT demonstrated rescued suppression of lipolysis we investigated insulin regulation of the lipolytic pathway only in these two fat pads. rWAT and iWAT exhibited enhanced AKT phosphorylation after insulin stimulation in LFD, PEP2 and ROSI but not in HFD groups, with an overall effect of insulin in both fat pads, but a dietary overall effect only in iWAT (Figure 3D,E and Figure S7).

#### 3.2.5. PPARγ Activation, Adipocyte Size and Adipogenesis Markers

**Figure 4:**
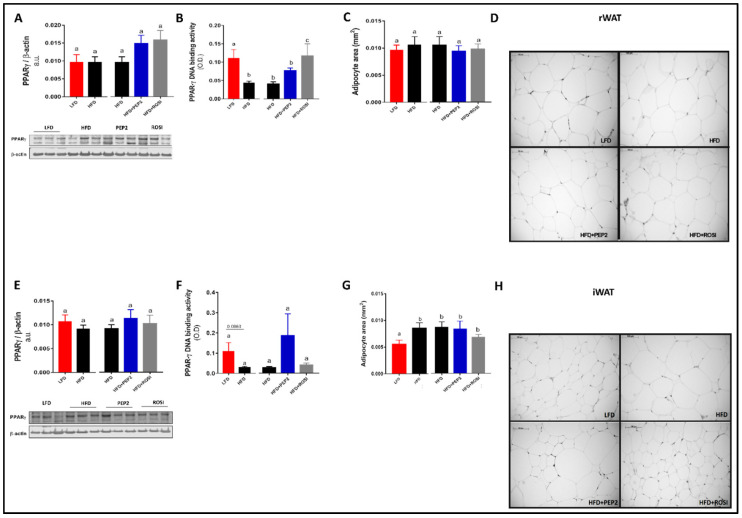
Retroperitoneal white adipose tissue (rWAT) and inguinal white adipose tissue (iWAT) adipogenesis markers in tissues harvested from mice treated with Peptide 2 (Protocol 2). (**A**) PPARγ protein abundance in rWAT; (**B**) PPARγ DNA binding activity in rWAT (n = 5–6); (**C**) adipocytes mean area in rWAT(n = 4); (**D**) representative image in rWAT; (**E**) PPARγ protein abundance in iWAT; (**F**) PPARγ DNA binding activity in iWAT (n = 5–6); (**G**) adipocytes mean area in iWAT (n = 4); (**H**) representative image in iWAT. Data expressed as mean ± SEM and analyzed by two tailed t-test (LFD vs. HFD) and by one-way ANOVA or Kruskal–Wallis (HFD groups). Bars with different letters indicates *p* < 0.05. HFD, high fat diet; LFD, low fat diet; rWAT, retroperitoneal WAT; eWAT, epididymal WAT; iWAT, inguinal WAT; PPARγ, peroxisome proliferator activated receptor gamma; ROSI, rosiglitazone.

Despite being increased 40–50% in PEP2 and ROSI groups, PPARγ protein abundance in rWAT did not reach statistical significance (Figure 4A). However, PPARγ activation was increased in LFD and ROSI groups compared to HFD, but not in PEP2 (Figure 4B). Image analysis revealed no differences in average adipocyte size or distribution in the rWAT (Figure 4C and Figure S8, respectively) in any diet group. In iWAT, total PPARγ protein abundance was similar between groups (Figure 4E) and PPARγ activation was not different between groups (Figure 4F). Average adipocyte size in LFD group was smaller than HFD (Figure 4, left). However, no changes were seen among the HFD groups (Figure 4G, right). The distribution curve showed reduced percentage of larger adipocytes (>0.017 mm^2^) in the LFD compared to HFD (Figure S8). Protein abundance of adipogenesis markers of adipogenesis, including adiponectin, perilipin-1, fatty-acid binding protein 4 and fatty acid synthase were similar between HFD groups in both rWAT and iWAT (Figure S8). In addition, we tested the effect of Peptide 2 during the differentiation of pre-adipocytes derived from both subcutaneous (9 W) and brown (9 B) adipose tissue pads from mice. However, no major effects were observed in terms of adipogenesis, lipolysis, lipogenesis and WAT browning (Figures S9 and S10).

#### 3.2.6. Liver Characterization

**Figure 5:**
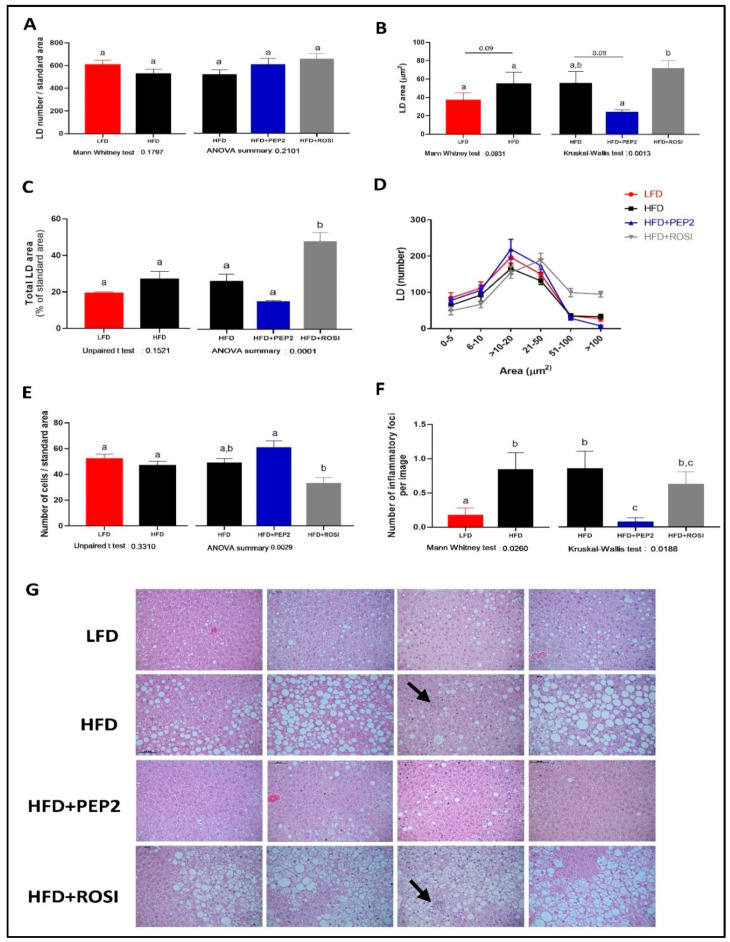
Liver characterization in tissues harvested from mice treated with Peptide 2 (Protocol 2). (**A**) total LD number (n = 6); (**B**) average individual LD area (n = 6); (**C**) average area covered by LD (n = 6); (**D**) LD distribution by size (n = 6); (**E**) number of cells per liver area analyzed (n = 6); (**F**) average inflammatory foci per image (n = 5–6); (**G**) liver representative images. Data expressed as mean ± SEM and analyzed by two tailed t-test (LFD vs. HFD) and by one-way ANOVA or Kruskal–Wallis (HFD groups). Arrows indicate inflammatory foci. Bars/lines with different letters indicates *p* < 0.05. LD, lipid droplet; HFD, high fat diet; LFD, low fat diet; ROSI, rosiglitazone.

Morphological characterization of LD showed no differences between LFD and HFD groups in terms of total LD number (Figure 5A, left) and total LD area (Figure 5C, left), but LFD had 30–40% smaller individual LD area (*p* = 0.09) (Figure 5B, left). Among HFD groups, despite a similar number of LD in all the groups (Figure 5A, right), ROSI had a similar individual LD area to HFD and an increased total LD area (Figure 5B,C, right, respectively). PEP2 had smaller individual LD area compared to ROSI, and around 50% smaller area compared to HFD (*p* = 0.09) (Figure 5B, right). In addition, PEP2 had 40–50% smaller total LD area compared to HFD, but while the ANOVA showed a *p* = 0.0001 suggesting an overall treatment effect, no statistical difference was observed between these groups (Figure 5C, right, *p* = 0.2). Albeit not statistically significant, the qualitative analysis of the images reveals that the majority of the animals had visibly less liver fat (first, second and fourth panel on Figure 5G) while the minority did not (third panel Figure 5G). These differences are also reflected by the distribution curve (Figure 5D), which emphasizes the right-shift in LD area as well as more abundant LD > 50 μm^2^ in area in the ROSI livers. Regarding hepatocyte size, no difference between LFD and HFD was observed, but PEP2 had a higher number of cells per area than ROSI, indicative of less hypertrophy (Figure 5E, right). Moreover, PEP2 and LFD exhibited fewer inflammatory foci compared to HFD, while ROSI had an intermediate effect (Figure 5F, right). The differences seen in hepatic TG content (Table 3) and LD characterization are supported by the representative images, where we observed smaller LD, less area covered in LD and reduced inflammatory foci presence in LFD and PEP2 groups, while ROSI exhibited most of the image covered in LD (Figure 5G). Hepatic cholesterol content was lower in the PEP2 group compared to HFD and ROSI groups, but not different between LFD and HFD (Table 3). Plasma ALT was not different among the HFD groups, but LFD had lower plasma ALT concentration than HFD animals (Table 3). Collagen staining to identify fibrosis revealed no presence of collagen within the hepatic parenchyma in almost all of the samples. However, all the samples from ROSI group had at least 1 image out of 19 with presence of weak collagen staining. One sample in the LFD group exhibited marked collagen staining, which was attributed to a random finding of fibrosis (Table S3).

#### 3.2.7. Liver PPARγ and AT2R and Insulin Signaling

**Figure 6:**
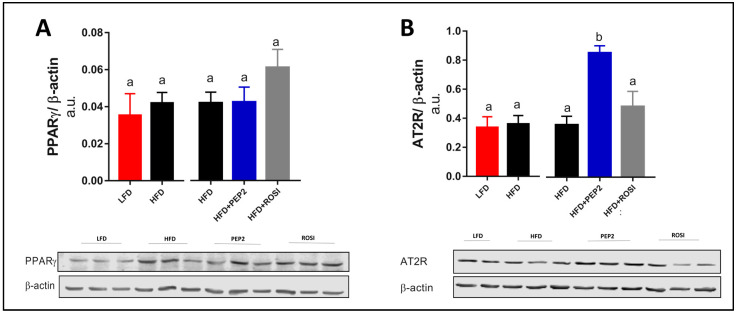
Liver protein abundance in tissues harvested from mice treated with Peptide 2 (Protocol 2). (**A**) PPARγ protein abundance and (**B**) AT2R. Data expressed as mean ± SEM of n = 5–6 mice. Data analyzed by two-tailed t-test (LFD vs. HFD) and by one-way ANOVA or Kruskal-Wallis (HFD groups). Bars with different letters indicates *p* < 0.05. PPARγ, peroxisome proliferator-activated receptor gamma; AT2R, angiotensin II type 2 receptor.

Hepatic PPARγ abundance was similar between all groups (Figure 6A). Interestingly, angiotensin II-type 2 receptor (AT2R) protein abundance was enhanced 2-fold (*p* < 0.05) in the PEP2 group compared to HFD (Figure 6B, right).

#### 3.2.8. Lipid Metabolism, Inflammation, and Fibrosis Genes

**Figure 7:**
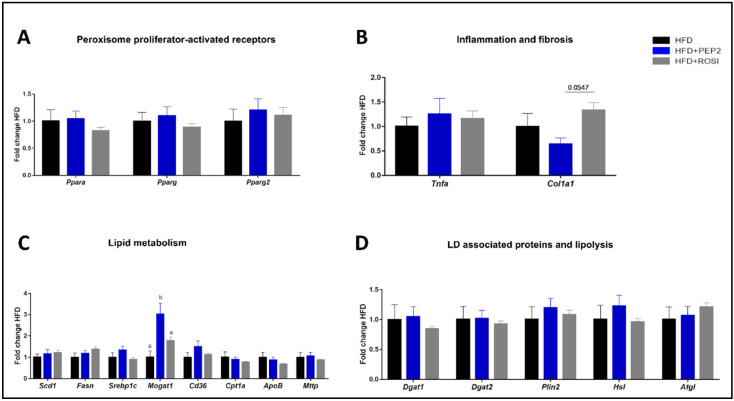
Liver gene expression in tissues harvested from mice treated with Peptide 2 (Protocol 2). (**A**) Peroxisome proliferator-activated receptors; (**B**) inflammation and fibrosis related genes; (**C**) lipid metabolism related genes and (**D**) LD associated proteins and lipolysis related genes. Data expressed as mean ± SEM of fold change of HFD control (n = 7–8) and analyzed by one-way ANOVA or Kruskal–Wallis test. Data normalized to β-actin gene expression. Bars with different letters indicates *p* < 0.05. LD, lipid droplet.

mRNA expression of PPPAR alpha (*Ppara*), gamma (*Pparg*) and gamma2 (*Pparg2*) was not different among the HFD groups (Figure 7A). Although pro-inflammatory gene Tnfa was not different among the HFD groups, a trend of reduced *Col1a1* (*p* = 0.055), a marker of fibrosis, was observed in PEP2 compared with ROSI (Figure 7B). Among the several genes involved in lipid metabolism analyzed, only *Mogat1* showed a significant difference, with a 3-fold increase in the PEP2 group compared to HFD, and an approximately 1.5-fold increase compared to ROSI (Figure 7C). No changes were seen in selected genes that encode LD-associated proteins or that are involved in lipolysis (Figure 7D). No difference in any of the analyzed genes was observed between LFD and HFD (Figure S11).

## 4. Discussion

The prevalences of NAFLD, obesity and T2D are increasing in parallel. In fact, because of the relationship between hepatic steatosis and metabolic diseases, there is a movement to change the parameters used for diagnosis of NAFLD and a name change to metabolic-associated fatty liver disease (MAFLD) is also proposed [24]; however, we have used NAFLD as the chosen abbreviation. Bioactive peptides have potential to aid in the management of metabolic diseases because they can modulate physiological processes [9] either in conjunction with pharmacological treatments or as novel, stand-alone approaches for conditions without approved pharmacotherapy, such as NAFLD. In this study, we hypothesized that diet supplementation with Peptide 2 would improve systemic and local IR and as a consequence, modulate features of NAFLD in HFD-induced obese-IR mice. We found that Peptide 2 supplementation: (1) improved systemic IR during the hyperinsulinemic-euglycemic clamp; (2) rescued insulin-regulated lipolysis in rWAT and iWAT, despite no change in adipocyte size or BW; (3) reduced hepatic lipid accumulation while increasing monoacylglycerol O-acyltransferase 1 (*Mogat1*) gene expression in the liver; and (4) reduced hepatic inflammatory infiltration.

EWH is a mixture of bioactive peptides shown before to improve IR and reduce adipocyte size in rodents [11]. Herein, we showed that PPARγ activation is enhanced in rWAT of EWH supplemented animals, which was accompanied by a better response to insulin in terms of suppression of enzymes involved in lipolysis in rWAT as well as increased p-AKT [11]. Another possibility is that HFD reduced baseline p-HSL/HSL ratio, which was restored by EWH leading to the insulin suppression of p-HSL observed. Nevertheless, these findings led us to hypothesize that the metabolic improvements seen in vivo were due to modulation of PPARγ in WAT, similar to the action of thiazolidinediones, which would induce adipogenesis and promote the appearance of more insulin sensitive adipocytes [25] improving systemic insulin sensitivity. We then asked if there were specific peptides from the EWH mixture that could modulate PPARγ abundance and an in vitro screening revealed that a few peptides were able to mimic insulin effect of enhancing PPARγ in preadipocytes [15], which led us to investigate their effects in vivo. The peptide with greater potential based on our screening (Figure S3) was Peptide 2 and we further evaluated its effects in obese and IR mice focusing on WAT and the liver.

IR is a key feature of T2D, and it plays a significant, multi-factorial role in the development of NAFLD [26]. IR in WAT leads to impaired suppression of lipolysis, which increases NEFA delivered to the liver whereas hepatic IR impairs the suppression of gluconeogenesis [26]. Egg-derived peptides and hydrolysates were shown before to improve insulin sensitivity in rodents [11,14,27] but relative contributions of hepatic versus non-hepatic tissues were not evaluated. Therefore, we first investigated the potential of Peptide 2 supplementation to reduce IR using a hyperinsulinemic-euglycemic clamp, the gold standard technique for this outcome measurement. We observed that LFD, PEP2 and ROSI groups had lower IR compared to HFD group. Although rosiglitazone, a known insulin sensitizer, was expected to reduce IR, this is the first study showing that dietary supplementation with Peptide 2 improves IR, despite no changes in glucose tolerance or BW. Moreover, suppression of endogenous glucose production during clamp was stronger in the PEP2 and LFD groups compared to HFD, consistent with better hepatic insulin sensitivity. This improvement in IR is supported by a reduced HOMA-IR in the LFD group, but despite a decrease in PEP2 group HOMA-IR, it was not statistically significant. Not only was systemic insulin sensitivity was improved by Peptide 2 supplementation but WAT insulin signaling was also rescued. The normalized regulation of lipolysis by insulin would be predicted to reduce NEFA delivery to the liver. Taken together, these results suggests that the improvement in systemic insulin sensitivity in the PEP2 group could be in part because of a decreased spillover of lipids from WAT to non-adipose tissues due to a better hormonal regulation of lipolysis.

We then asked if the improvement in WAT insulin signaling was associated with PPARγ activation. PPARγ regulates adipogenesis and has an important role in glucose and lipid metabolism. We previously showed that Peptide 2 increased PPARγ protein in adipocyte cell culture [15]. However, we did not see higher PPARγ activation in any of the fat pads tested. This is accompanied by no overall changes in WAT adipocyte size or adipogenesis markers among HFD group, substantiated by no effect in adipogenesis markers during preadipocyte differentiation of mouse-derived cells in culture. On the other hand, we observed increased activation of PPARγ in rWAT of ROSI animals. Therefore, the effects of Peptide 2 are different from rosiglitazone, supporting a PPARγ independent mechanism of action. We conclude at this point that Peptide 2 acting to improve insulin signaling independently of PPARγ, at least in the models used in this study; therefore, other mechanisms need to be investigated.

Ongoing efforts seek to validate the use of insulin sensitizers, such as thiazolidinediones for the treatment of NAFLD. However, findings are controversial, with most of the benefits seen with pioglitazone rather than rosiglitazone [7,8]. In rodents, rosiglitazone plays a dual role depending on the level of hepatic PPARγ expression [28]. For example, in mice with low hepatic expression of PPARγ, rosiglitazone protects against lipid accumulation while in obese mice with elevated PPARγ, rosiglitazone exacerbates hepatic steatosis [28]. In fact, PPARγ is a key up-regulator of hepatic steatosis in HFD-induced obese mice treated with rosiglitazone [29]. Similarly, in this study we show that the ROSI group, although more insulin-sensitive, exhibits higher liver weight and TG content compared to HFD, whereas PEP2 does not worsen HFD-induced hepatic steatosis. PPARγ is mainly expressed in adipose tissue, but it is also expressed in the liver [30] and its expression is enhanced in the liver of HFD fed animals [28,29]. Similarly to the literature, in our study HFD feeding tended to increase hepatic Pparg2 gene expression and protein compared to the LFD group, but neither PEP2 nor ROSI groups affected *Pparg* or *Pparg2* mRNA. However, albeit not significant, we saw a trend to increased hepatic PPARγ protein abundance in the ROSI group compared to HFD control. Peptide 2 supplementation did not increase PPARγ protein abundance above the HFD-induced effect. Although we did not measure hepatic PPARγ DNA binding activity, the results suggest that Peptide 2 may not be directly activating hepatic PPARγ, suggesting a difference in mechanism from rosiglitazone.

In NAFLD, progression to NASH is characterized by increased LD size, hepatocyte hypertrophy and inflammation [2,5]. Surprisingly, the ROSI group exhibited a true microvesicular steatosis mixed with macrovesicular steatosis, while PEP2 and HFD groups appeared to have macrovesicular steatosis, which is considered more pathological [20,21,22]. However, PEP2 tended to decrease LD area compared to HFD. Therefore, PEP2 group presented a macrovesicular steatosis with small LD vs. macrovesicular steatosis with large LD in the HFD group. The importance of LD size is highlighted by findings showing that macrovesicular steatosis is linked to fibrosis and microcirculation impairment in rodents [31,32] and fibrogenesis in humans [20]. Moreover, the extent of macrovesicular steatosis can impact liver transplantation and graft survival in humans [21]. Other food-derived peptides reduce LD size, for example pepsin-generated EWH supplementation reduces the number and size of LD in rats, accompanied by a decrease in plasma inflammatory and oxidative stress markers [33]. In addition, supplementation with a peptide derived from sweet lupine (GPETAFLR) improves hepatic steatosis and reduces TG content. The mechanism of action suggested was through PPARα and uncoupling protein 1 (UCP1) activation and reduced hepatic expression of fatty-acid synthase gene (*Fasn*) and inflammatory markers, but only mRNA levels were measured, not their activity [34]. In another study, the improvement in NAFLD seen after potato-derived peptide (DIKTNKPVIF) supplementation is accredited to adenosine monophosphate-activated protein kinase (AMPK) activation and decreased inflammatory markers [35]. We find no changes in gene expression of *Ppara* or *Fasn* after Peptide 2 supplementation. Therefore, the mechanism behind the effect of PEP2 supplementation to modulate hepatic LD size is unclear. Decrease in BW is associated with improvements in NAFLD; being an important confounding factor when evaluating an interventional study. Howewer, we observed modulation of LD size by Peptide 2 independently of changes in BW. In the studies mentioned above one of them reported similar final BW but a lower eWAT mass [33], while the other reported decreased BW [34] and one did not report BW measurements [35]. It is worth noting that several peptides reduce hepatic lipid accumulation; however, whether it is a protein (amino-acid)-related or a peptide-specific effect still remains to be determined.

The observed improvement in liver morphology was independent of hepatic TG content, which was not significantly different between PEP2 and HFD groups, but a decrease in cholesterol content was seen between HFD and PEP2 groups, similar to Song et al. [36], who reports a reduction of the hepatic total cholesterol content after quinoa supplementation but no difference between HFD and quinoa supplemented groups in terms of hepatic TG [36] and attributes the improvement in NAFLD in part to changes in hepatic phospholipids, such as increased lysophosphatidylcholine and pantothenic acid, and decreased phosphatidylcholine and dioleoylphosphatidylcholine [36]. Indeed, impairment of cholesterol metabolism may be the key driver to large LD formation, rather than TG metabolism [20]. Despite higher hepatic TG content in the ROSI group, lack of change in plasma ALT indicates no further liver damage on top of that induced by HFD. Therefore, rather than only LD size or amount, its lipid composition also plays a key role in causing hepatic damage.

Inflammation and fibrosis are important markers to evaluate NAFLD progression to NASH. Peptide 2 supplementation attenuated the density of hepatic inflammatory foci consistent with the parent EWH hydrolysate decreasing plasma inflammatory markers. In the same study, an increase in AT2R was seen in WAT and liver [11]. Moreover, the hydrolysate reduced blood pressure in rats by modulating the renin-angiotensin system (RAS), including reduction of angiotensin II-type 1 receptor and induction of AT2R abundance in the aorta of rats [10]. AT2R is not only involved in the modulation of blood pressure by opposing angiotensin II-type 1 receptor activity, but its stimulation is associated with reno- and cardio-protective effects, anti-inflammatory and antifibrotic action, among others as extensively reviewed elsewhere [37]. In fact, RAS modulation is linked to hepatic fibrosis in rodents and humans with NAFLD [38,39]. In addition, AT2R has antifibrotic action in the liver of mice [40] and plays a role in blood flow regulation [41,42]. When we evaluated the presence of collagen staining as an indication of fibrosis, we found no presence of collagen in most of the samples. This may be because 14 wk of 45% HFD is insufficient to induce marked inflammation and fibrosis. Although *Col1a1* mRNA was increased after 19 wk of 60% HFD, only at 50 wk of dietary intervention was fibrosis seen in histological analysis [43]. Considering that macrovesicular steatosis with large LD impairs hepatic blood flow [31] and our finding that PEP2 had the highest hepatic AT2R and lowest mRNA expression of type 1 collagen, we speculate that AT2R up-regulation may be a mechanism by which PEP2 decreases inflammation and fibrosis over a longer-term of NAFLD but further research is needed to confirm this speculation. Conversely, ROSI did not induce AT2R or suppress collagen gene expression, differentiating its hepatic effects from PEP2.

Many pathways underlie hepatic lipid accumulation including increased delivery of NEFA to the liver following WAT lipolysis, decreased fatty acid oxidation, increased DNL or decreased VLDL secretion [2]. Although differences in gene expression involved in glucose and lipid metabolism occur between hepatocytes displaying large and small LD in the liver [44], surprisingly the mRNA expression patterns are similar between ROSI and HFD groups. This does not exclude the role of ROSI in modulating lipid metabolism pathways since we did not measure the activity of the related enzymes. The only difference observed between PEP2 and HFD group was higher *Mogat1* gene expression in the PEP2 group. *Mogat1* encodes the mannosylglycoprotein N-acetyl-glucosaminyl transferase 1 (MGAT1), which catalyzes TG synthesis via the monoacylglycerol O-acyltransferase pathway. The role of MGAT1 in liver steatosis is controversial, with some studies showing that silencing of hepatic MGAT1 improves steatosis and blood glucose levels [45,46]. Conversely, others demonstrate that MGAT1 knockout in liver does not improve hepatic steatosis, liver TG content, insulin sensitivity or glucose tolerance in HFD-fed mice [47]. Moreover, hepatic overexpression of MGAT1 does not increase liver TG content in HFD mice, but does in LFD animals [47]. In our study using fasted animals, we found reduced LD size concomitant with higher *Mogat1* gene expression, but no other direct target of PPARγ had increased expression. Intriguingly, increased hepatic *Mogat1* expression and MGAT activity occur after prolonged fasting with higher fat oxidation, and are both dependent and independent of *Ppara* expression, suggesting that MGAT1 regulates the hepatic fasting response [48]. This is consistent with the reduced LD size found in our study. We did not see altered *Ppara* gene expression, and we did not measure its activity directly, therefore, further investigation is needed to elucidate the role of Peptide 2 in hepatic lipid metabolism and its relationship to MGAT1.

Because our intended use of rosiglitazone was as a positive control for insulin sensitization and PPARγ agonism, differences in the hepatic phenotype versus PEP2 were unexpected. Our results suggest that, in contrast to rosiglitazone, Peptide 2 does not activate PPARγ and, in conditions of HFD-induced obesity and IR, Peptide 2 does not worsen HFD-induced hepatic steatosis. Rather, Peptide 2 supplementation improves IR and rescues insulin-regulated lipolysis in WAT while tending to reduce LD area, decreasing inflammation, and possibly preventing fibrosis, crucial processes to prevent NAFLD progression to NASH.

We note that an HFD containing 45% kcal fat is not commonly used to generate NASH animal models, with most of the diet-induced models receiving a high fat/high sugar diet. Thus, less hepatic inflammation and fibrosis in our model was observed compared with the available literature [49,50]. The peptide was administered mixed in the animals’ diet, which does not allow for precise specification of the dose of peptide received by each animal. On the other hand, this dietary intervention has advantages over daily gavage for chronic studies in terms of reducing stress in the animals, which worsens IR, our main outcome. It is important to note that oxidative stress can also be involved in hepatic steatosis progression to NASH and that several food-derived peptides exert antioxidant activity as recently reviewed [51]. In future research, it would be worth exploring the antioxidant activity of Peptide 2 as a possible mechanism of preventing disease progression. In addition, it would be relevant to test Peptide 2 bioavailability and absorption pathways, including ability to modulate cell junction proteins. Lastly, all of our data reflect the overnight fasting state which, compared to fed state, may yield a different gene expression pattern and less pronounced effects in some of the outcomes reported.

In conclusion, this study shows for the first time that Peptide 2 diet supplementation improves IR in HFD-induced obese and IR mice, while at the same time preventing further exacerbation of HFD-induced NAFLD features independently of BW. On the other hand, rosiglitazone-treated mice, despite having improved IR, exhibited worse hepatic steatosis if administered together with HFD. Therefore, compared to rosiglitazone, Peptide 2 promotes more beneficial effects on the combined outcomes of insulin resistance, WAT dysfunction and hepatic steatosis.

## Figures and Tables

**Table 1:** Peptide 2 specifications.

	Peptide 2
Amino acid sequence	QAMPFRVTEQE
Number of amino acids	11
Theoretical molecular weight (g/mol) *	1335.50
Observed molecular weight (g/mol)	1335.8
Theoretical isoelectric point *	4.53
Grand average of hydropathicity (GRAVY) *	−0.918
Hydrophobicity *	22
Terminus modifications	None
Net charge at pH 7.0 *	−1

* Parameters calculated using online tools: ProtParam (Expasy); Bachem peptide calculator and Thermofischer Peptide analyzing tool. Observed molecular weight provided by Genscript.

**Table 2:** Resistin and adiponectin concentration in plasma, epididymal and retroperitoneal adipose tissue in Sprague Dawley rats treated with HFD+4% EWH for 6 weeks (Protocol 1). Data expressed as mean ± SEM and analyzed by two tailed t-test (n = 6–7).

	HFD	HFD + 4% EWH
Plasma (fasting)		
Resistin (pg/mL)	1047 ± 84.55	1011 ± 82.99
Adiponectin (ng/mL)	55,853 ± 2832	60,270 ± 7367
eWAT		
Resistin (pg/mL)	175.1 ± 15.67	166.5 ± 11.14
Adiponectin (ng/mL)	1002 ± 28.44	1044 ± 19.54
rWAT		
Resistin (pg/mL)	172.3 ± 20.14	175.1 ± 15.67
Adiponectin (ng/mL)	1033 ± 25	1026 ± 28.73

Abbreviations: HFD, high fat diet; EWH, egg white hydrolysate; eWAT, epididymal adipose tissue; rWAT, retroperitoneal adipose tissue.

**Table 3:** Body weight, body composition and plasma profile of C57BL/6 mice at the end of the Peptide 2 feeding trial (Protocol 2). Data expressed as mean + SEM and analyzed by two-tailed t-test (LFD × HFD) and by one-way ANOVA or Kruskal-Wallis (HFD groups). Different letters on the same row indicates *p* < 0.05 for HFD groups. # Indicates *p* < 0.05 and ^ indicates *p* < 0.1 compared to HFD.

	LFD	HFD	HFD + PEP2	HFD + ROSI
Body composition (not fasted)				
Initial BW (g) (week 6)	29.1 ± 0.6 ^#^	33.9 + 0.7 ^a^	32.9 + 0.6 ^a^	33.5 + 0.7 ^a^
Final BW (g)	33.4 ± 0.7 ^#^	42.5 ± 0.7 ^a^	41.4 ± 0.5 ^a.b^	39.4 ± 0.8 ^b^
BW gain (%) (week 6–13)	15.4 ± 1.0 ^#^	25.0 ± 1.3 ^a^	26.2 ± 1.2 ^a^	17.7 ± 1.5 ^b^
Final fat mass (% BW)	25.6 ± 1.3 ^#^	38.5 ± 1.1 ^a^	38.6 ± 0.6 ^a^	36.1 ± 1.1 ^a^
Final lean mass(% BW)	65.9 ± 1.2 ^#^	54.2 ± 1.0 ^a^	54.3 ± 0.6 ^a^	56.5 ± 1.0 ^a^
Tissue weight (g/BW)				
eWAT	0.039 ± 0.0029 ^#^	0.059 ± 0.0029 ^a^	0.061 ± 0.0014 ^a^	0.057 ± 0.0025 ^a^
rWAT	0.017 ± 0.00079 ^#^	0.028 ± 0.00084 ^a^	0.025 ± 0.00096 ^a,b^	0.022 ± 0.0014 ^b^
iWAT	0.046 ± 0.0070 ^#^	0.073 ± 0.0049 ^a^	0.065 ± 0.0027 ^a^	0.062 ± 0.0040 ^a^
Liver	0.036 ± 0.0019	0.033 ± 0.0015 ^a^	0.032 ± 0.0007 ^a^	0.044 ± 0.0015 ^b^
Plasma (fasting)				
Glucose (mmo/L)	4.5 ± 0.3 ^#^	5.9 ± 0.3 ^a^	5.3 ± 0.3 ^a^	5.6 ± 0.2 ^a^
Insulin (ng/mL)	0.5 ± 0.1 ^	1.0 ± 0.1 ^a^	0.9 ± 0.1 ^a^	0.6 ± 0.1 ^a^
HOMA-IR	0.8 ± 0.2 ^#^	2.4 ± 0.6 ^a^	1.8 ± 0.3 ^a^	1.9 ± 0.3 ^a^
NEFA (mEq/L)	0.4 ± 0.02	0.4 ± 0.02 ^a^	0.5 ± 0.03 ^a^	0.5 ± 0.04 ^a^
TG (mg/dL)	39.4 ± 5.6	52.6 ± 5.8 ^a^	43.2 ± 4.2 ^a^	41.9 ± 4.6 ^a^
Plasma ALT (mU/mL)	8.4 ± 4.0 ^#^	28.4 ± 27.1 ^a^	24.9 ± 15.1 ^a^	20.9 ± 13.5 ^a^
Liver content (mg/g tissue)				
TG	46.8 ± 2.3 ^#^	71.5 ± 11.0 ^a^	57.6 ± 6.7 ^a^	132.7 ± 12.1 ^b^
Cholesterol	2.4 ± 0.2	2.1 ± 0.2 ^a^	1.3 ± 0.1 ^b^	1.8 ± 0.1 ^a^

Abbreviations: ALT, alanine aminotransferase; BW, body weight; eWAT, epidydimal white adipose tissue (WAT); HFD, high fat diet; HOMA-IR, homeostatic model assessment of insulin resistance; iWAT, inguinal WAT; LFD, low fat diet; NEFA, non-esterified fatty acids; rWAT, retroperitoneal WAT; TG, triglycerides.

## Data Availability

Data are contained within the article or Supplementary Materials.

## Supplementary Materials

The following supporting information can be downloaded at: https://www.mdpi.com/article/10.3390/metabo13020174/s1, Table S1: diet composition; Table S2: primers sequence; Table S3: fibrosis assessment; Figure S1: high performance liquid chromatography and mass spectrometry of Peptide 2; Figure S2: EWH effects in WAT of Sprague Dawley; Figure S3: ITT and tissues weight for the in vivo peptide screening; Figure S4: Food and water intake; Figure S5: OGTT before peptide treatment; Figure S6: hyperinsulinemic-euglycemic clamp and ITT; Figure S7: adipose tissue total AKT and p-AKT; Figure S8: adipogenesis markers; Figure S9: Effect of PEP2 on 9 W pre-adipocyte differentiation; Figure S10: Effect of PEP2 on 9 B pre-adipocyte differentiation; Figure S11: lipid metabolism gene expression in LFD and HFD groups.
