# Supplementary material for: An Egg White-Derived Peptide Enhances Systemic Insulin Sensitivity and Modulates Markers of Non-Alcoholic Fatty Liver Disease in Obese, Insulin Resistant Mice
Source: Metabolites. 2023 Jan 24;13(2):174. doi: 10.3390/metabo13020174 (PMC9965836; doi:10.3390/metabo13020174)
Supplement: Supplementary file 1 [file metabolites-13-00174-s001.zip › metabolites-2176183-supplementary.pdf]

SUPPLEMENTARY MATERIALS

Supplementary table S1. Diet composition.

	**LFD**	**HFD**	**HFD+PEP2**	**HFD+ROSI**
**Casein** (g/Kg)	210.0	245.0	245.0	245.0
**L-Cystine** (g/Kg)	3.0	3.5	3.5	3.5
**Corn Starch** (g/Kg)	445.0	85.0	85.0	85.0
**Maltodextrin** (g/Kg)	50.0	115.0	115.0	115.0
**Sucrose** (g/Kg)	160.0	200.0	200.0	200.0
**Lard** (g/Kg)	20.0	195.0	195.0	195.0
**Soybean Oil** (g/Kg)	20.0	30.0	30.0	30.0
**Cellulose** (g/Kg)	37.15	58.0	58.0	58.0
**Mineral Mix, AIN-93G-MX (94046)** (g/Kg)	35.0	43.0	43.0	43.0
**Calcium Phosphate, dibasic** (g/Kg)	2.0	3.4	3.4	3.4
**Vitamin Mix, AIN-93-VX (94047)** (g/Kg)	15.0	19.0	19.0	19.0
**Choline Bitartrate** (g/Kg)	2.75	3.0	3.0	3.0
**PEP2** (mg/Kg BW)	n/a	n/a	45.0	n/a
**Rosiglitazone** (μM/Kg BW in water)	n/a	n/a	n/a	2.5

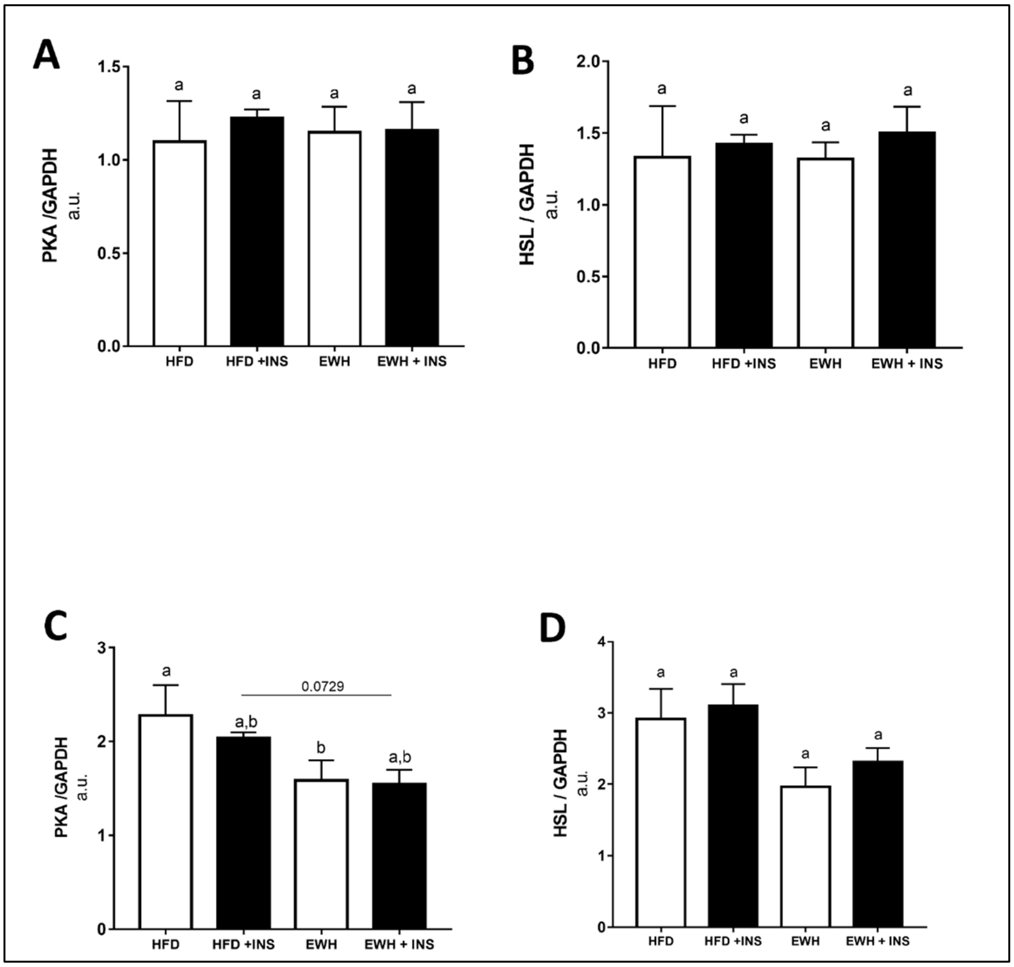

Figure S1. EWH effects in WAT of Sprague Dawley. PKA, p-PKA, HSL and p-HSL protein abundance in rWAT (A-B) and eWAT (C-D). Data expressed as mean $\pm$ SEM and analyzed by one-way ANOVA (n=3-4). Bars with different letters indicates $p \leq 0.05$. EWH, egg white hydrolysate; PKA, protein kinase A; HSL, hormone sensitive lipase; WAT, white adipose tissue; rWAT, retroperitoneal WAT; eWAT, epididymal WAT.

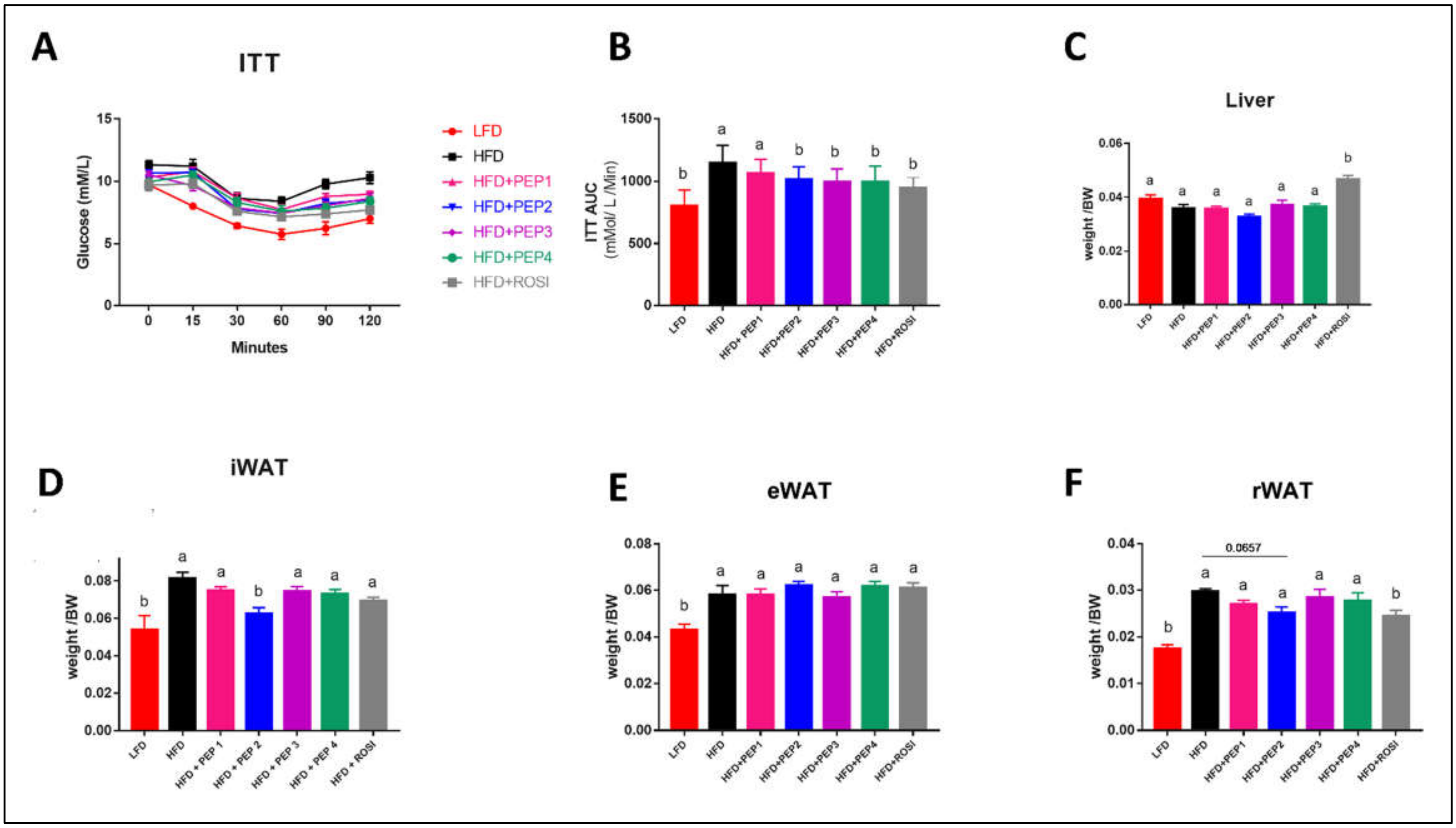

Figure S2. (A-B) Insulin tolerance test (ITT) and (C-F) tissue weight after supplementation with 4 different peptides or rosiglitazone. (A) ITT, (B) ITT area under the curve (AUC), (C) rWAT, (D) iWAT, (E) eWAT and (F) liver. Data expressed as mean $\pm$ SEM and analyzed by two-way ANOVA (A) or one-way ANOVA with HFD group set as control. (A-C, E) n=11-12, (D) n=9-12 and (F) n=7-12. Bars with (b) indicates $p \leq 0.05$ compared to HFD group. WAT, white adipose tissue; rWAT, retroperitoneal WAT; eWAT, epididymal WAT; iWAT, inguinal WAT.

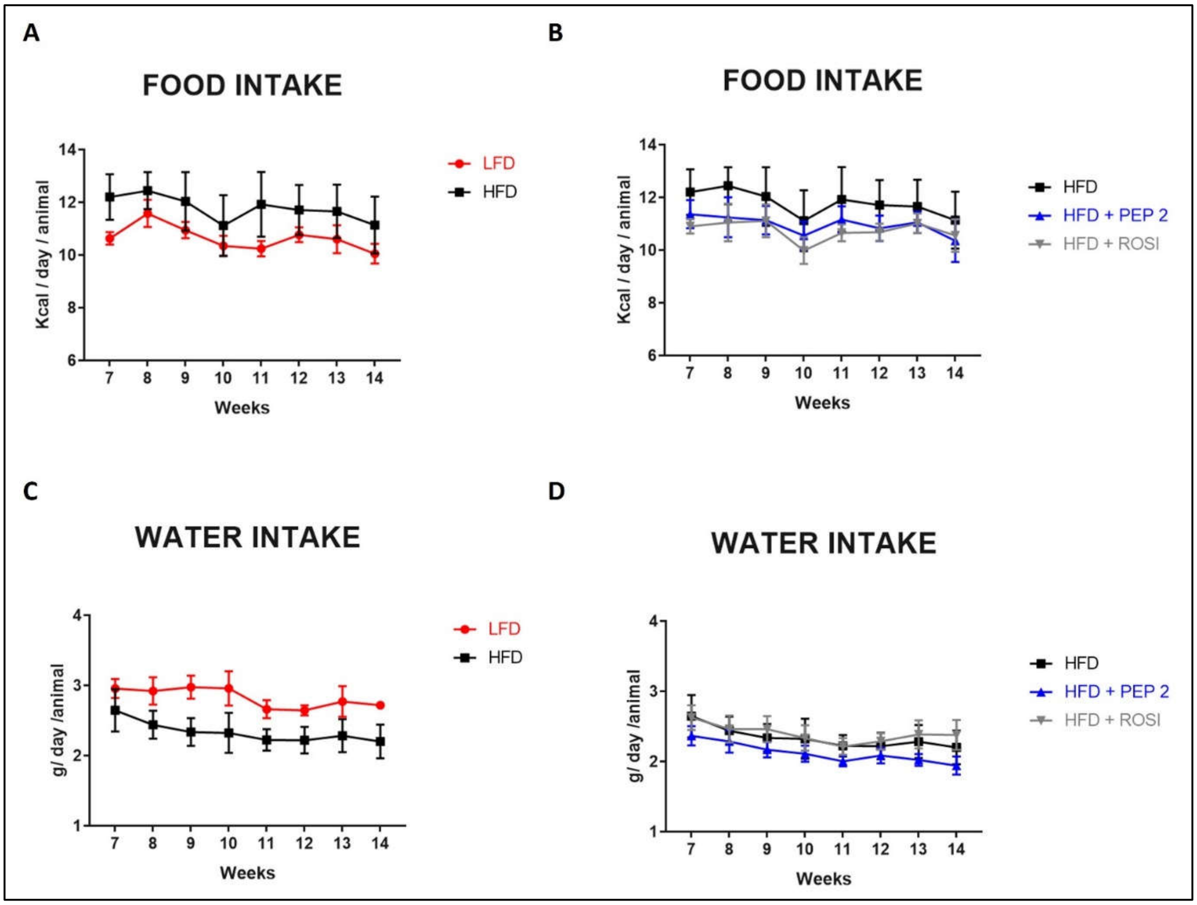

Figure S3. (A and B) Food and (C and D) water intake during diet supplementation period. Data expressed as mean ± SEM and analyzed by one-way ANOVA. HFD, high fat diet; LFD, low fat diet; ROSI, rosiglitazone.

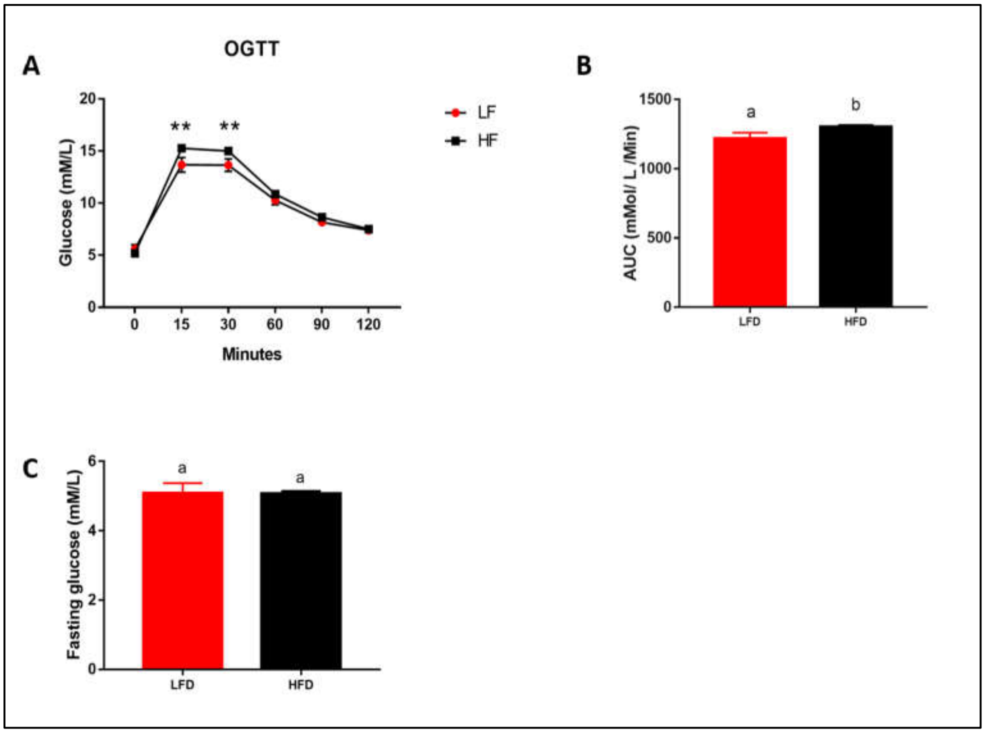

Figure S4. (A) OGTT, (B) area under the curve (n=12 LFD and 69 HFD) and (C) fasting glucose(n=20 LFD and 101 HFD) after 6 weeks of HFD feeding. Data expressed as mean ± SEM and analyzed by two-way ANOVA (A) or two-tailed t-test (B and C). Bars with different letters indicates $p \leq 0.05$. ** $p<0.01$. HFD, high fat diet; LFD, low fat diet.

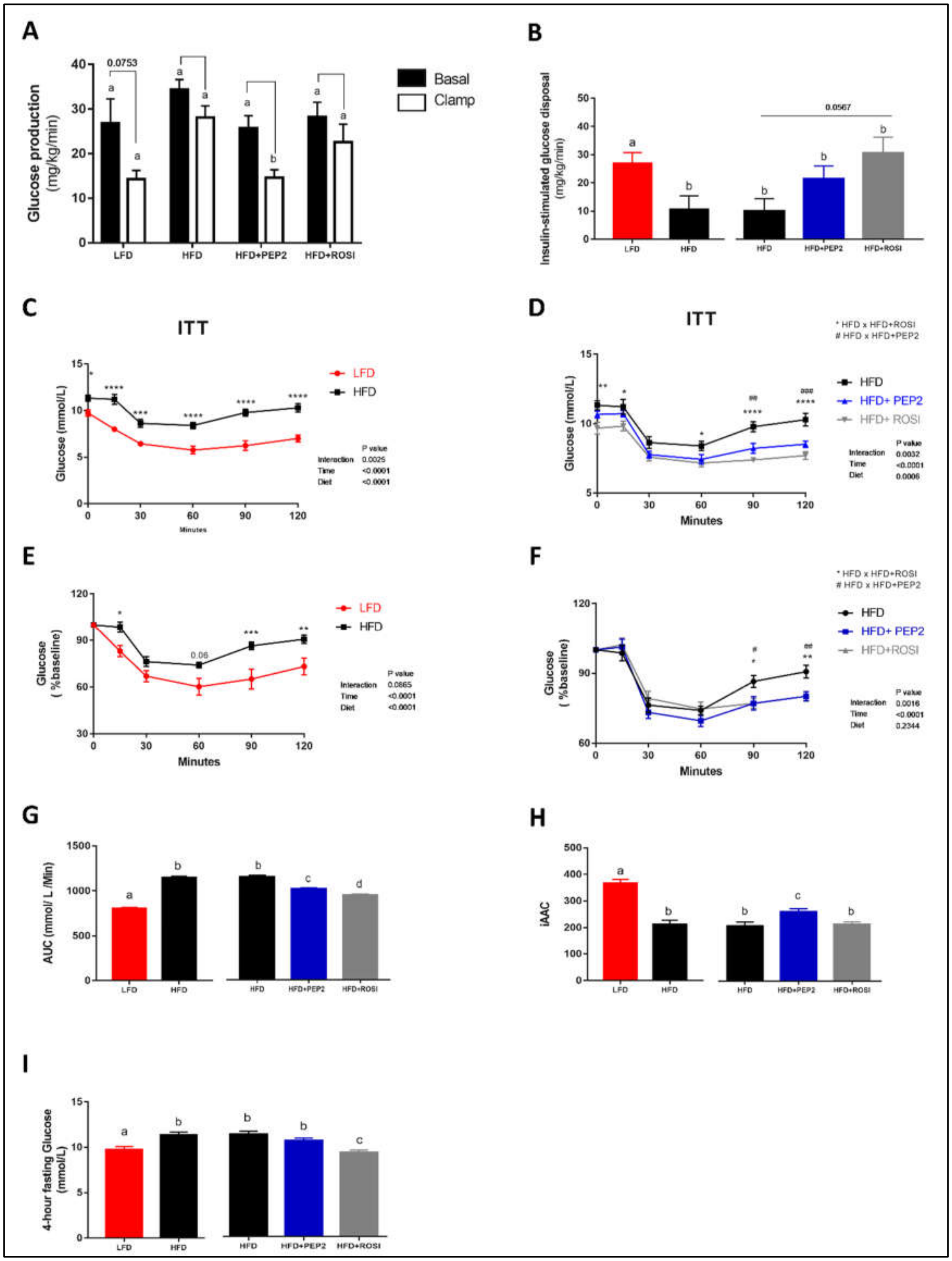

Figure S5. Hyperinsulinemic-euglycemic clamp and Insulin tolerance test (ITT). (A) within group glucose production comparison at basal state and during clamp; (B) insulin stimulated glucose disposal. Data expressed as mean ± SEM. Figure A and B (LFD x HFD) was analyzed by two tailed t test and figure B (HFD groups) analyzed by one-way ANOVA (n=4-7). (C/D) ITT LFD x HFD and ITT HFD groups (n=11-12); (E/F) insulin tolerance test (ITT) as % of baseline blood glucose concentration (n=11-12), (G) ITT AUC, (H) ITT incremental area above the curve (AAC) and (I) 4-hour fasting glucose on ITT day (n=12). Data expressed as mean ± SEM and analyzed by two-way ANOVA (C-F) and by two tailed t test (LFD x HFD) or by one-way ANOVA (HFD groups) (G-I). * Indicates $p \leq 0.05$, $^{**}p<0.01$, $^{***}p<0.001$ $^{****}p<0.0001$. HFD, high fat diet; LFD, low fat diet; ROSI, rosiglitazone. BW, body weight; HFD, high fat diet; LFD, low fat diet; ROSI, rosiglitazone. Bars with different letters indicates $p \leq 0.05$.

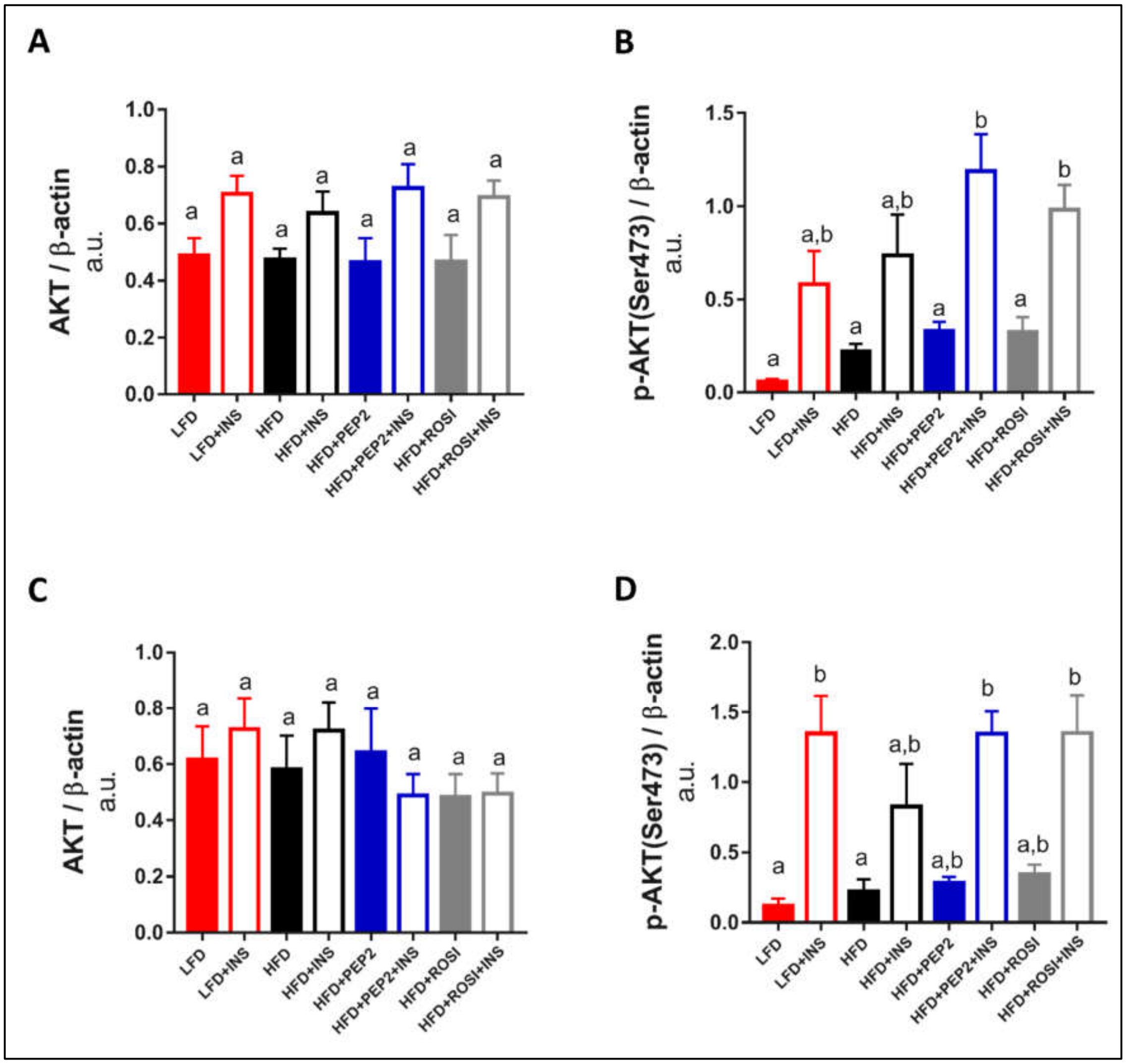

Figure S6. (A) rWAT AKT/β-actin; **(B)** rWAT p-AKT/ β-actin; **(C)** iWAT AKT/β-actin; **(D)** iWAT p-AKT/β-actin**.** Data expressed as mean ± SEM of n=6 mice. Data analyzed by one-way ANOVA or Kruskal-Wallis. Bars with different letters indicate p≤0.05. HFD, high fat diet; LFD, low fat diet; rWAT, retroperitoneal WAT; eWAT, epididymal WAT; iWAT, inguinal WAT; ROSI, rosiglitazone.

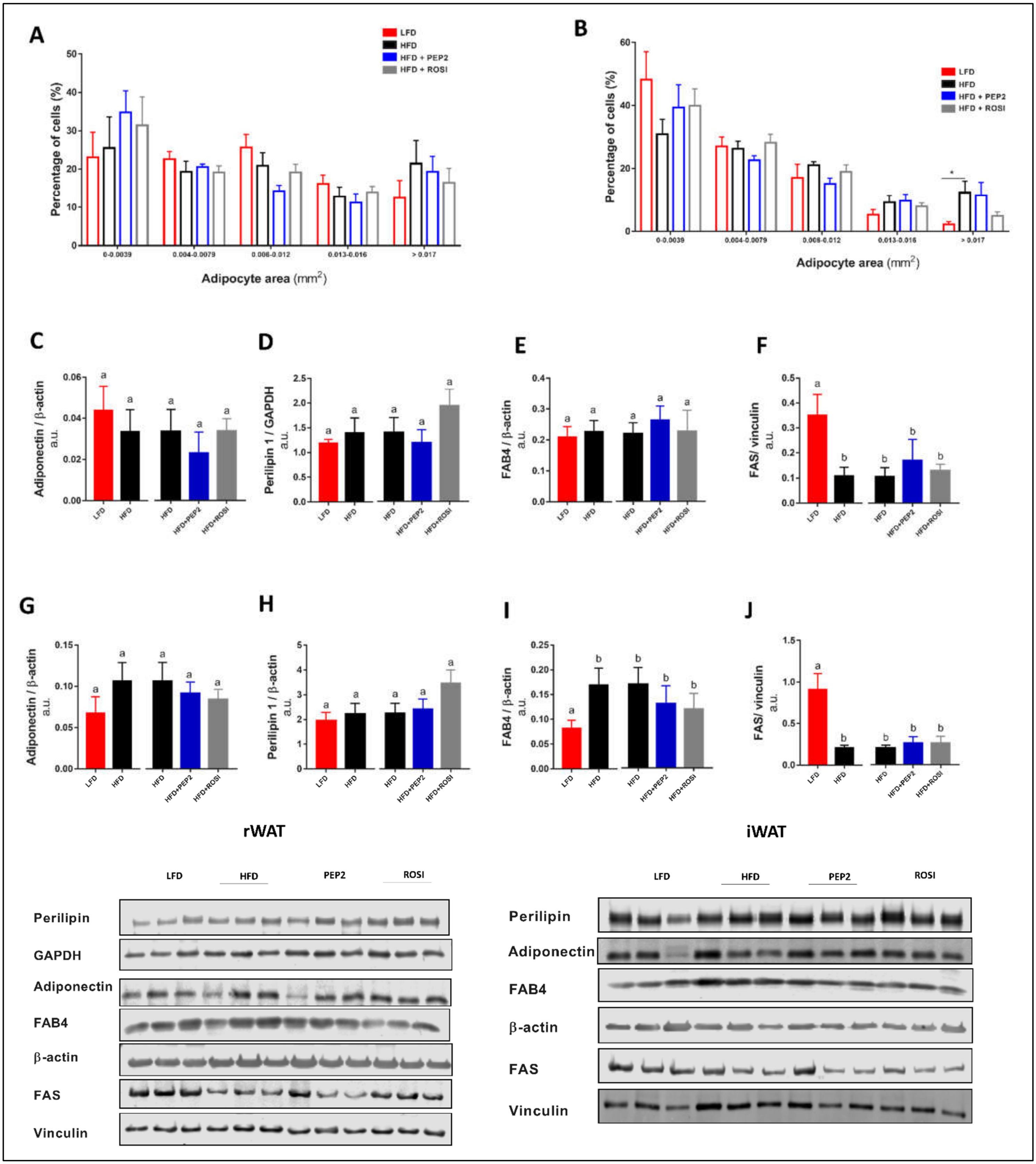

Figure S7. Adipogenesis markers western blot. (A) adipocyte size distribution in rWAT (n=4) and (B) adipocyte size distribution in iWAT (n=4). (C) Adiponectin rWAT, (D) Perilipin-1 rWAT, (E) Fatty acid binding protein-4 (FAB4)) rWAT, (F) Fatty-acids synthase (FAS) rWAT, (G) Adiponectin iWAT, (H) Perilipin-1 iWAT, (I) Fatty acid binding protein-4 (FAB4)) iWAT, (J) Fatty-acids synthase (FAS) iWAT. Data expressed as mean $\pm$ SEM of n=5-6 mice and analyzed by two-tailed t-test (LFD x HFD) or by one-way ANOVA or Kruskal-Wallis (HFD groups). Bars with different letters indicates $p<0.05$. HFD, high fat diet; LFD, low fat diet; rWAT, retroperitoneal WAT; eWAT, epididymal WAT; iWAT, inguinal WAT; ROSI, rosiglitazone.

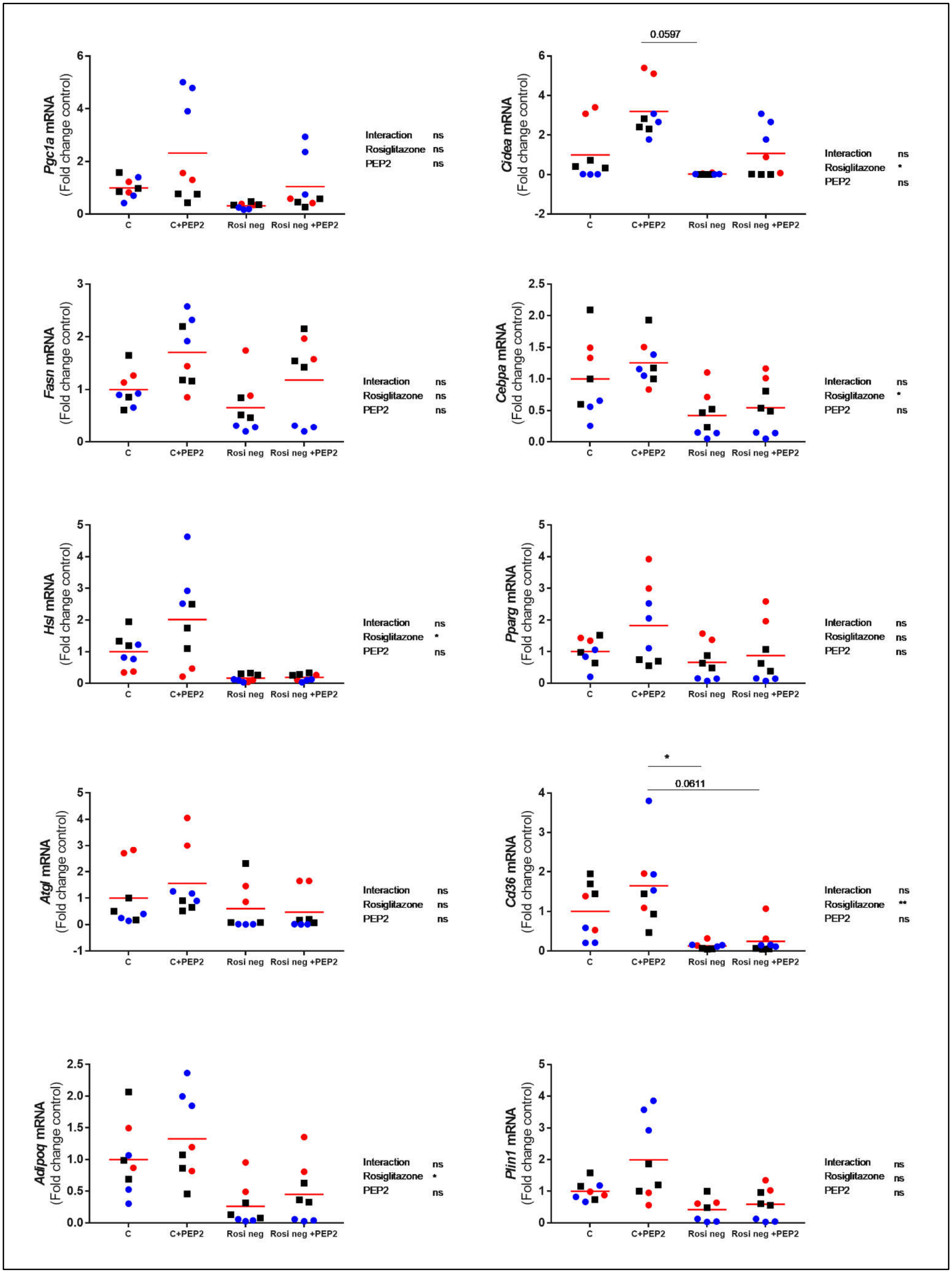

Figure S8. Effect of PEP2 on 9W pre-adipocyte differentiation. Data expressed as grand mean of fold change compared to control. Data analyzed by two-way ANOVA. Different color indicates biological replicates.

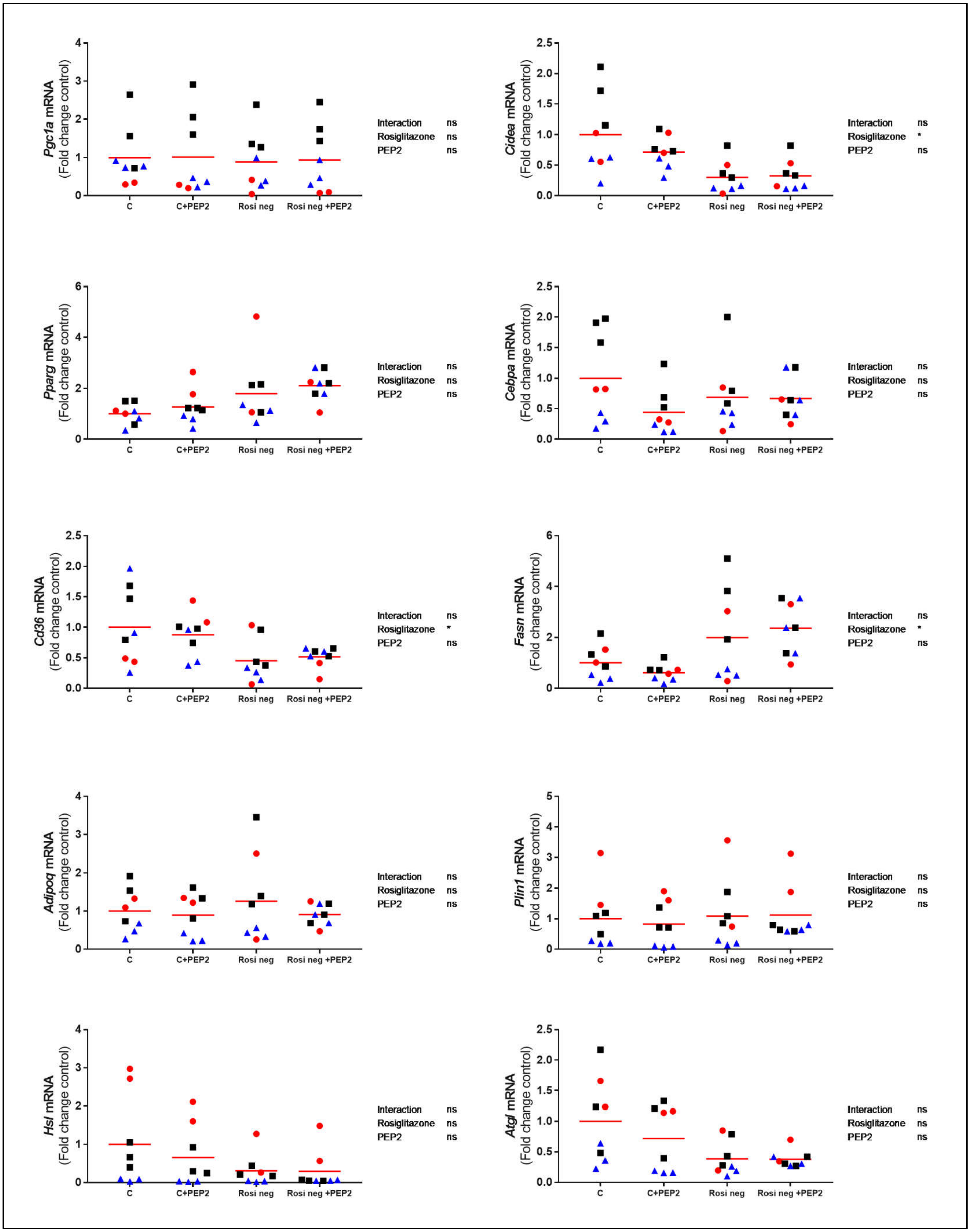

Figure S9. Effect of PEP2 on 9B pre-adipocyte differentiation. Data expressed as grand mean of fold change compared to control. Data analyzed by two-way ANOVA. Different color indicates biological replicates.

Table S2. Primers sequences. *Gene symbol* followed by its (name) and the encoded protein: *Ppara* (peroxisome proliferator-activated receptor alpha) encodes PPARα; *Pparg* (peroxisome proliferator-activated receptor gamma) encodes PPARγ; Pparg2 (peroxisome proliferator-activated receptor gamma) encodes PPARγ2; *Tnfa* (tumor necrosis factor alpha) encodes TNF-α; *Col1a1* (collagen type 1 alpha 1 chain) encodes pro-alpha1 chains of type 1 collagen; *Scd1* (stearoyl-Coenzyme A desaturase 1) SCD1; *Fasn* (fatty acid synthase) encodes FAS; *Srebp1c* (sterol regulatory element binding transcription factor 1c) encodes SREBP-1c; *Mogat1* (monoacylglycerol O-acyltransferase 1) encodes MGAT1; *Cd36* encodes fatty acid translocase (CD36); *Cpt1a* (carnitine palmitoyl transferase 1A) encodes CPT1α; *ApoB* (apolipoprotein B) encodes ApoB; *Mttp* (microsomal triglyceride transfer protein) encodes MTP; *Dgat1* (diacylglycerol O-acyltransferase 1) encodes DGAT1; *Dgat2* (diacylglycerol O-acyltransferase 2) encodes DGAT2; *Plin2* (perilipin 2) encodes PLIN2; *Hsl* (hormone sensitive lipase) encodes HSL; *Atgl* (patatin-like phospholipase domain containing 2) encodes adipose triglyceride lipase (ATGL).

			**amplicon size**
TNF-a	F 5' – 3'	GGTTCTCTTCACGGGACAAGGC	100
	R 5' – 3'	AGAGAGGAGGTTGACTTTCTCCTG	
CD36	F 5' – 3'	TGGCTAAATGAGACTGGGACC	126
	R 5' – 3'	ACATCACCACTCCAATCCCAAGTAAGG	
Fasn	F 5' – 3'	CTTCCGTCACTTCCAGTTAGAGCAG	179
	R 5' – 3'	AGTTCAGTGAGGCGTAGTAGACAGTG	
Mogat1	F 5' – 3'	CCTTGACCCATGGTGCCAGTT	108
	R 5' – 3'	CGTCTTGTATAGTTCGTAGCCAGGAGC	
Ppparg	F 5' – 3'	GAAGACATTCCATTCACAAGAGCTGACC	142
	R 5' – 3'	GCCTGTTGTAGAGCTGGGTCTT	
Ppparg 2	F 5' – 3'	ATGCTGTTATGGGTGAAACTCTGGGA	152
	R 5' – 3'	CACAGAGCTGATTCCGAAGTTGGTG	
Ppara	F 5' – 3'	CGACCTGAAAGATTCGGAAACTGCAG	182
	R 5' – 3'	GCGTCTTCTCGGCCATACACAAG	
Srebp1c	F 5' – 3'	GGAGCCATGGATTGCACATTTGAAGACAT	180
	R 5' – 3'	TTCCAGAGAGGAGGCCAGAGA	
Cpt1a	F 5' – 3'	CCTACCATGGCTGGATGTTTGCAG	154
	R 5' – 3'	GTATCTTTGACAGCTGGGACAGGCA	
SCD1	F 5' – 3'	GTTCCCTCCTGCAAGCTCTACAC	121
	R 5' – 3'	GCAGCCGTGCCTTGTAAGTTC	
Dgat2	F 5' – 3'	CTGGCAAGAACGCAGTCA	161
	R 5' – 3'	TTCTTCTGGACCCATCGG	
β-actin	F 5' – 3'	AGCTATGAGCTGCCTGACG	106
	R 5' – 3'	TGCCACAGGATTCCATACCCAAG	
Atgl	F 5' – 3'	TGTGGCCTCATTCCTCCTAC	158
	R 5' – 3'	TCGTGGATGTTGGTGGAGCT	
Hsl	F 5' – 3'	GCT GGG CTG TCA AGC ACT GT	160

	R 5' – 3'	GTA ACT GGG TAG GCT GCC AT	
Plin2	F 5' – 3'	CTGCGGCCATGACAAGT	123
	R 5' – 3'	GCTGGTTCAGAATAGGCAGTCTTT	
Dgat1	F 5' – 3'	GAGTCTATCACTCCAGTGGG	100
	R 5' – 3'	GGCGGCACCACAGGTTGACA	
ApoB	F 5' – 3'	ACTGTGACTTCAATGTGGAG	150
	R 5' – 3'	CTGAGGCAGACAGACTTGTC	
Col1a1	F 5' – 3'	TCAGACCTGTGTGTTCCCTACT	119
	R 5' – 3'	ACGGGAATCCATCGGTCAT	
Mttp	F 5' – 3'	AGAGGACAGCTTTGTCACCG	113
	R 5' – 3'	TCTTCAGCTCCAATTTCTGCTTCG	

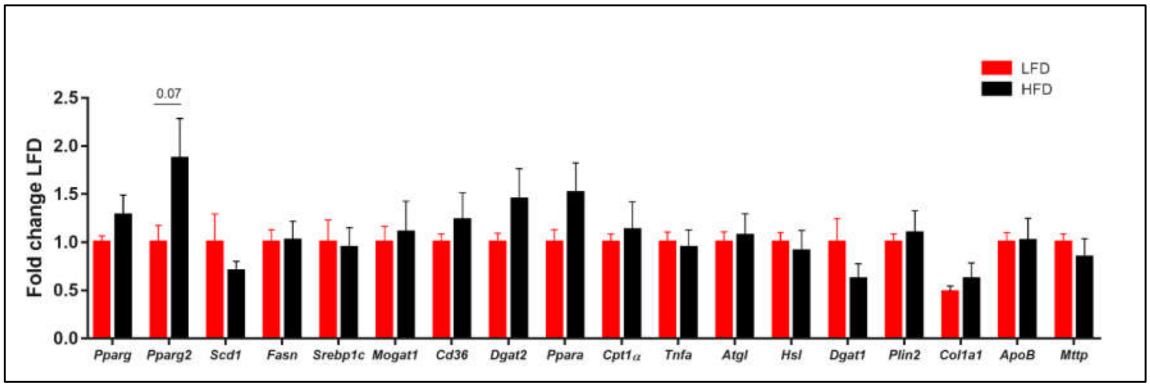

Figure S10. Lipid metabolism gene expression in LFD and HFD groups. Data expressed as mean ± SEM of fold change of LFD control and analyzed by two tailed t test (n=7). Data normalized to β-actin gene expression. HFD, high fat diet; LFD, low fat diet.

Table S3. Fibrosis assessment.

GROUPS	Number of images POSITIVE FOR FIBROSIS (REVIEWER 1)	Number of images POSITIVE FOR FIBROSIS (REVIEWER 2)	FINAL AVERAGE
**LFD-29**	0/19	0/19	0/19
**LFD-31**	0/19	0/19	0/19
**LFD-F6**	17/19	19/19	18/19
**HFD-35**	0/19	0/19	0/19
**HFD-63**	0/19	0/19	0/19
**HFD-F17**	1/19	0/19	0.5/19
**PEP2-43**	0/19	0/19	0/19
**PEP2-F16**	0/19	0/19	0/19
**PEP2-F35**	0/19	2/19	1/19
**ROSI-25**	0/19	1/19	0.5/19
**ROSI-27**	0/19	0/19	0/19
**ROSI-53**	1/19	0/19	0.5/19
